# Quantifying the impact of strong ties in international scientific research collaboration

**DOI:** 10.1371/journal.pone.0280521

**Published:** 2023-01-17

**Authors:** Junwan Liu, Xiaofei Guo, Shuo Xu, Yueyan Zhang

**Affiliations:** College of Economics and Management, Beijing University of Technology, Beijing, People’s Republic of China; Pohang University of Science and Technology, REPUBLIC OF KOREA

## Abstract

Tie strength has been examined as an antecedent of creativity. Although it has been discovered that international collaboration affects scientific performance, the effect of tie strength in the international collaboration network has been largely neglected. Based on international publications of 72 countries/regions published from 1993 to 2013, we combine descriptive and panel regression methods to examine how the bonding of strong collaboration ties contributes to countries’ international scientific performance. Strong ties occur at an average rate of 1 in 4 collaborators, whereas countries/regions share on average 84% of articles with their strong-tie collaborators. Our quantitative results provide an explanation for this phenomenon in international collaboration: the establishment of a strong tie relationship contributes to above-average productivity and citation frequency for countries/regions. To further explore which types of strong ties tend to have stronger citation impact, we analyse the relationship between persistent and stable collaboration and publication citation impact. Experimental results show that international collaborations with greater persistence and moderate stability tend to produce high impact publications. It is noteworthy that when the collaboration period is divided into different time intervals, similar findings can be found after the same analysis procedure is carried out. This indicates that our conclusions are robust. Overall, this study provides quantitative insights into the added value of long-term commitment and social trust associated with strong collaborative partnerships in international collaboration.

## 1. Introduction

With the development of globalization, research activities have become increasingly internationalized, and scientific collaboration among countries has become an inexorable trend [1, 2]. Collaboration contributes to the exchange of ideas and enables the combination of different kinds of knowledge to create something novel and useful [3]. Many countries have formulated corresponding policies to promote international collaboration, hoping to develop both their national strength and international competitiveness [4]. In international collaboration, academic publications are considered as a significant output of scientific collaborative research activities. Many studies have observed the growth of international collaboration in academic sciences [5, 6], which has resulted in the emergence of the international collaboration network [7]. The international collaboration network is considered as a collection of ego networks, which are formed by egocentric networks linked according to copartners [8]. Egocentric collaboration networks depict the one-to-many relationship between an ego and its collaborative countries [9].

It is important to place a productive country or region within a network of collaborative relationships to better understand its participation in international collaboration [10]. Scholars have paid much attention to the role of collaboration ties in scientific collaboration networks [11, 12]. Collaboration ties are the scientific collaboration relationships that bind scientists together in a collaboration network, and tie strength represents the strength of the relationship [13]. The dichotomy between strong and weak ties is a long-standing research point in the research community [14]. It has been argued that weak ties (i.e., ties of low duration, infrequent interactions and low emotional closeness) provide access to distinct social circles and, thus, to diverse information, whereas strong ties (i.e., ties of long duration, frequent interactions and high emotional closeness) provide access and trust to similar individuals [14, 15]. Strong partnerships entail higher trust and reciprocity, which reduce the costs and risks associated with new collaborations [16].

The characteristics of collaboration ties shape collaboration networks, which, in turn, affect the impact or usefulness of the publications created by the network [17]. Previous studies have extensively investigated the effect of international collaboration on scientific research performance [18, 19], whereas, from a micro perspective, the effect of collaboration tie strength in the egocentric collaboration network has been largely neglected.

Against this background, this paper uses quantitative methods to enhance our understanding of the relationship between collaboration ties and scientific performance from the perspective of international collaboration. Specifically, we analyse the collaboration ties among 72 countries/regions from an egocentric perspective and focus on the effect of tie strength on citation impact and productivity at the national level. Moreover, from the perspective of international collaboration, the stability and persistence of strong ties are explored, as they can offer insights into which types of international collaborations are “optimal” and which types tend to have the strongest citation impact [20]. By combining persistence and stability, we can gain a better understanding on the structure of collaboration. For this purpose, we identify the following research questions:

- What kinds of ties exist across different countries/regions in terms of international research collaboration? Are they weak, strong or super ties?
- How do strong ties in international scientific collaborations affect productivity and the number of citations of articles?
- How do stability and persistence interact to influence the success (measured by citation impact) of international papers produced through strong-tie collaborations?

The remainder of this study is structured as follows. Section 2 presents an overview of the literature on the features of international collaboration. Section 3 introduces the data gathered for this study and the methods for quantifying and distinguishing international collaborative ties. Subsequently, the results are thoroughly analysed and discussed. Finally, conclusions are drawn from the analysis of the results, and policy suggestions are outlined.

## 2. Literature review

### 2.1 International scientific collaboration and its network structure

In the era of globalization, scientific collaboration among different countries has become a general trend [21]. Gazni et al. [22] observed a growth pattern in the number of co-authors per paper at the international level and in the average number of organizations and nations per publication. Wagner and Leydesdorff [5] attributed the rapid growth of international collaboration to self-organization or preferential attachment. Globalization increases countries’ interconnectedness and interdependence, and results in the emergence of an international collaboration network [23].

By analysing international paper collaboration networks from the Science Citation Index (SCI) database, Leydesdorff and Wagner [24] highlighted the notable “small world” characteristic of the global collaboration network. Gui et al. [25] pointed out that although traditional scientific powers occupy a central position in the international collaboration network, emerging scientific powers are increasing in term of influence, and all countries are promoting the evolution of the world order. Several researchers have emphasized the impact of the network structure on node performance [26, 27]. Guan et al. [28] showed that collaboration network structure had a positive effect on country-level R&D efficiency by using patent collaboration data from the United States Patent and Trademark Office (USPTO). Gui et al. [29] demonstrated that higher degree centrality, structural holes and a small world quotient would enhance national knowledge productivity. Although collaborations provide benefits to both researchers and countries, the construction of a collaboration network also entails significant costs [30, 31]. Hence, authors must make a tradeoff to maximize the utility of such collaborations [32].

### 2.2 Collaborative ties in scientific collaboration networks

By exposing collaborators to various knowledge and perspectives, collaboration can increase the chances of novel ideas being generated [33, 34]. International collaboration networks are shaped by collaborative relationships among countries. To study the impact of collaboration ties on national scientific research, we consider only direct collaboration ties and their strength, which play a much more important role in the creation of knowledge [35].

Tie strength can be defined as “a combination of the amount of time, the emotional intensity, the intimacy (mutual confiding), and the reciprocal services which characterize the tie” [14]. In scientific collaboration networks, individuals with similar goals form ties to maintain long-term partnerships [36]. Strong ties are related to dense network blocs, while weak ties provide the possibility of connecting these blocs [37]. Moreover, strong ties can promote knowledge transfer and creation [38, 39]. By quantifying collaboration strength and investigating weak, strong, and super ties in the scientific collaboration network, Peterson [36] pointed out that super-tie cooperation could increase not only author productivity but also the number of citations at the researcher level. Based on a quantification of strong ties, Coccia and Wang [40] studied the impact of collaboration on the scientific output of economists and suggested that their productivity depends on their willingness to collaborate, international contacts, and collaboration stability.

### 2.3 Impact of international collaboration on research performance

Compared to short-distance collaboration, long-distance collaboration is costlier to practitioners in terms of time and resources. As Barjak and Robinson [41] pointed out, international collaboration must provide additional benefits that outweigh transaction costs; otherwise, its impressive growth would be difficult to explain.

Most of the extant literature has argued that international collaborations and partnerships can provide rich opportunities to enhance research [42, 43]. Wagner and Leydesdorff [5] indicated that international collaboration tends to free scientists from local constraints, such as social contexts that affect intellectual agendas. Compared to research groups that are limited in terms of geographical areas, diverse views and experiences provide internationally connected research groups with a significant competitive advantage [18]. Both advanced and developing countries benefit from the globalization of science [21], meaning that collaboration is a “win–win” game. Ebersberger et al. [44] found that technological variety is positively associated with the propensity of individuals to collaborate with foreign partners. Smith et al. [45] found that publications with more countries in their affiliations performed better in eight disciplines between 1996 and 2012. Most of the extant literature holds a positive view of the contribution of collaborations to scientific output.

Overall, the existing literature has focused on the characteristics of macroscopic international collaboration networks and the impact of international collaboration on countries’ academic performance. Previous studies focused on the social capital that can be derived by a country from its position in the international network, whereas the effect of tie strength in the egocentric international collaboration network has been largely neglected. Tie strength involves only two collaborators. Therefore, tie strength can be more easily managed by individual countries, which allows for the drawing of more direct management recommendations and policies. From the perspective of egocentric collaboration networks at the national level, this study focuses on heterogeneity in collaborative tie strength and quantifies the added value of strong ties for countries’ scientific performance.

## 3. Materials and methods

### 3.1 Data

The dataset used in this study comes from the ArnetMiner academic social networking platform [46], which covers 2,092,356 academic articles from the field of computer science, published between 1936 and 2014, with 1,712,433 unique authors and 8,024,869 local citation relationships among these publications. ArnetMiner is an intelligence big data mining and service system platform that provides publicly available datasets for academic social network research. The data tags involving publish year, author’s name, author’s institution information are extracted. The citations received of each article are calculated based on the citation relationships recorded in ArnetMiner. Authors’ names are disambiguated according to Tang et al. [47]. Using the author’s institution information, the country information is extracted from the record. Unique text strings corresponding to a country’s name are ascertained within the author address tags to ensure that only one country is recognized from each address. After filtering with MySQL and Excel, we remove papers that lack complete country information.

Note that 1993 is taken as the analysis’s starting year in this study. In this way, data from countries like the Union of Soviet Socialist Republics, Yugoslavia, and Czechoslovakia are excluded from further analysis, since their names or composition have been changed since 1970. In addition, Germany was reunified in 1990, so using data from Germany after 1993 in our analysis also eliminates the ambiguity over the information of the country, which means that the data from the German Democratic Republic (1949–1990) is excluded from our analysis. The ArnetMiner dataset ends in 2014, and we pick 2013 as the ending year for analysis so that the papers published before 2013 can have a long-time window to accumulate citations. International collaborations are represented using coauthored papers with two or more countries/regions. In the end, we obtain a dataset of 153,545 international collaborative articles, which involves 114 countries/regions.

We employ the following counting procedures to calculate the number of international papers of each country/region. Regardless of the order of authorship, if collaborating authors’ institutions recorded in a paper are located in different countries/regions, each country/region is considered to have produced one paper. For example, if a paper has authors from two different countries/regions, those two countries/regions each receive one paper count. Using the thresholds of a minimum of 100 international collaboration papers, in Table 1, 72 countries/regions are selected as research objects.

**Table 1:** Ranking of countries/regions in term of the number of international publications.

Rank	Country/ Region	International Publications	Rank	Country/ Region	International Publications	Rank	Country/ Region	International Publications
1	USA	73329	25	Finland	2314	49	Hungary	516
2	Mainland China	41734	26	Brazil	2249	50	South Africa	494
3	UK	21996	27	New Zealand	2093	51	Qatar	481
4	Germany	19627	28	Norway	1927	52	Argentina	407
5	Canada	19434	29	Portugal	1731	53	Cyprus	367
6	Australia	14470	30	Turkey	1655	54	Lebanon	334
7	France	13274	31	Iran	1625	55	Bangladesh	327
8	Japan	11198	32	Poland	1511	56	Jordan	311
9	South Korea	9523	33	Mexico	1280	57	Slovenia	308
10	India	9304	34	Malaysia	1234	58	Algeria	296
11	Singapore	8574	35	Scotland	1108	59	Ukraine	289
12	Italy	8434	36	Saudi Arabia	1069	60	Serbia	258
13	Hong Kong	6449	37	Russia	911	61	Slovakia	242
14	Switzerland	5981	38	Romania	865	62	Bulgaria	222
15	Netherland	5915	39	Pakistan	857	63	Estonia	219
16	Spain	5739	40	Chile	798	64	Oman	187
17	Taiwan	4907	41	Czech Republic	775	65	Kuwait	177
18	Austria	4682	42	Egypt	770	66	Indonesia	165
19	Israel	3987	43	Thailand	709	67	Morocco	155
20	Belgium	3805	44	Colombia	603	68	Cuba	150
21	Sweden	3562	45	Vietnam	593	69	Macau	145
22	Ireland	2800	46	Luxembourg	592	70	Venezuela	136
23	Denmark	2646	47	Tunisia	569	71	Croatia	134
24	Greece	2574	48	The United Arab Emirates	536	72	Iceland	103

**Fig 1:**
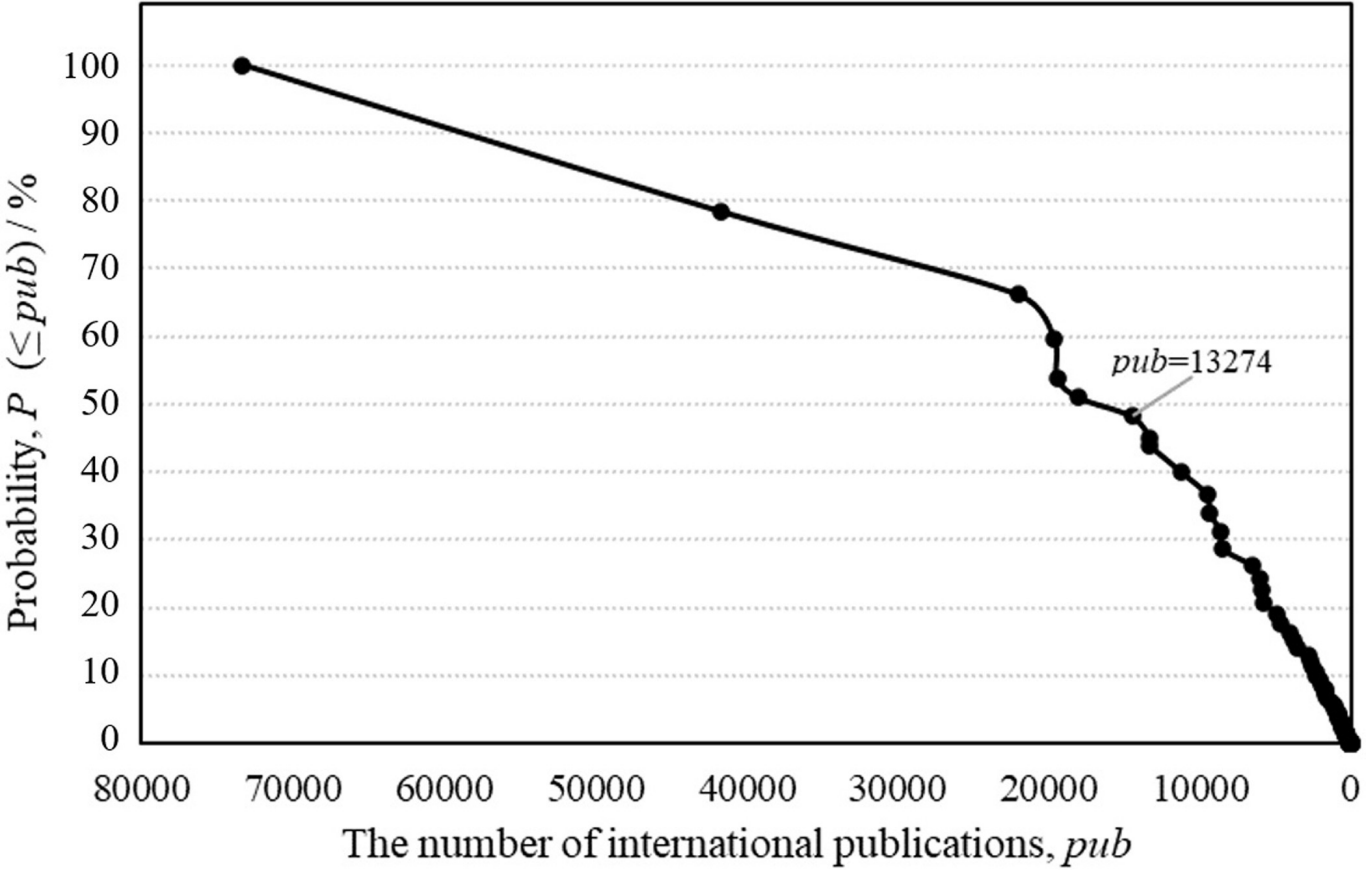
The cumulative distribution of the number of international publications.

In order to conduct a comparative analysis, we divide the 72 countries and regions into two groups (i.e., high- and low-productivity countries) according to the cumulative distribution of the total number of published international publications (Fig 1).

We take the point in Fig 1 which meet the following criteria to determine the high-productivity countries: (a) the point is the elbow point in the curve of cumulative distribution; (b) for countries at this point and before, the total number of international papers is greater than or equal to 50% of the total. We discover that the second inflection point, which represents the country with the seventh-highest number of international publications, fits the condition. We therefore refer to the top seven countries in terms of the quantity of published international papers as high-productivity countries, which are US, Mainland China, UK, Germany, Canada, Australia and France. The remaining countries/regions are referred to as low-productivity countries.

**Fig 2:**
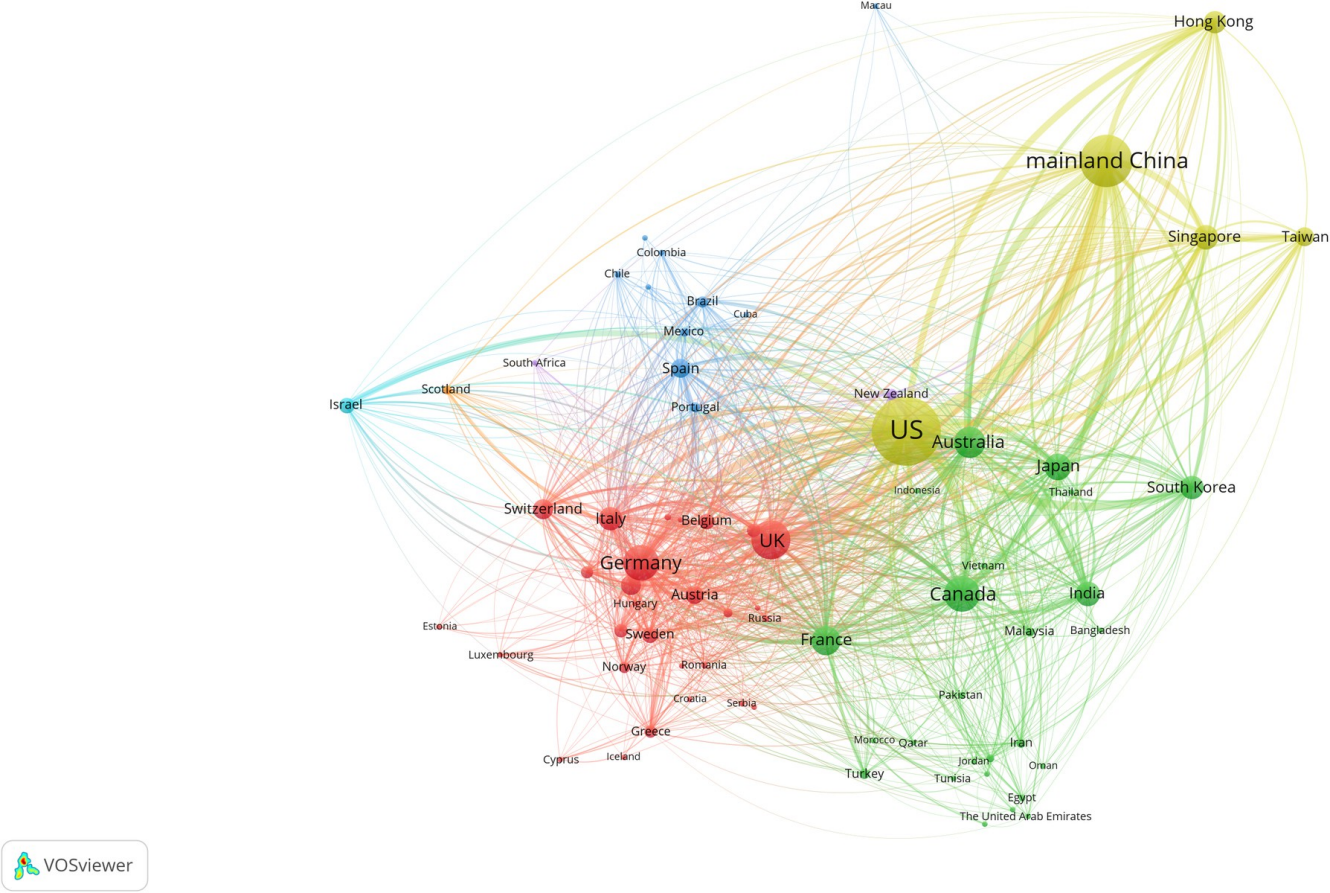
International scientific collaboration network in 1993–2013.

The international scientific collaborative network offers a unique perspective on the ranks and positions of countries/regions. We use VOSviewer to visualize the collaboration network of the 72 countries/regions (Fig 2). Each node in Fig 2 is sized according to the number of countries/regions collaborating in scientific research, indicating the degree node centrality. For example, the US ranks first and has the largest number of international publications, indicating that it cooperates with many countries in the scientific network and that other countries have strong interests in cooperating with it in scientific research. The node connection size is proportional to the frequency of paper collaboration between countries/regions, and different colours indicate different clusters.

The average path length of the network in Fig 2 is 1.30, which means that the collaboration network offers excellent accessibility. In order to study the international collaboration at various time slices, we divide the collaboration period into two stages, 1993–2003 and 2004–2013, and the resulting collaboration networks are illustrated in the S1 Appendix. In both S1 and S2 Figs in S1 Appendix, the US is the country with the most international collaborations. The average paths of the collaboration networks in different stages are 1.57 and 1.32, respectively. The clustering coefficients of the two collaboration networks are 0.77 and 0.83, respectively.

### 3.2 Research framework and methods

**Fig 3:**
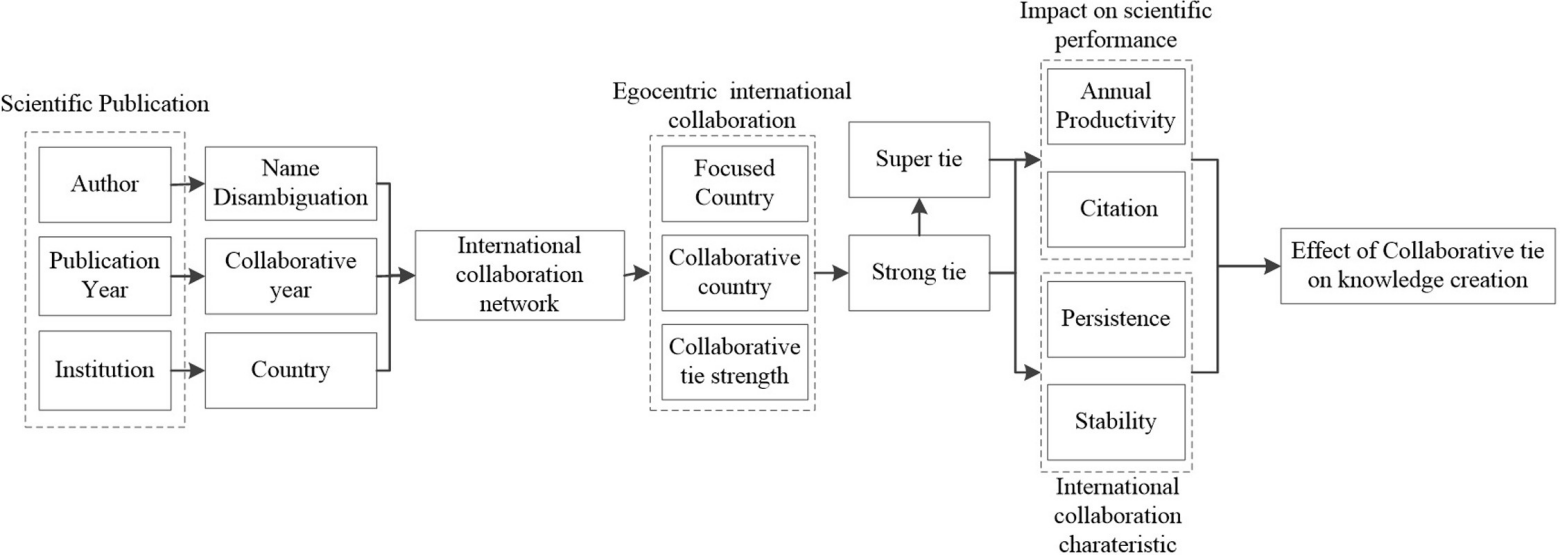
Research framework of this study.

As shown in Fig 3, our research framework consists of four phases. First, from the perspective of egocentric collaboration among countries/regions, we quantify the collaborative tie strength of the different collaboration relationships of each country/region. When we use a country/region as the subject of our analysis, we refer to it as the focused country/region since every country serves as the hub of its own egocentric collaborative network. Second, we investigate the role played by strong ties in international collaboration and explore the benefits that they provide to countries in terms of scientific research performance. Third, to investigate which types of international strong ties tend to result in high citation impact, we measure the persistence and stability of collaboration for each country pair with strong ties and explore how these two characteristics interact to influence scientific performance. Finally, to lay the groundwork for future research, we quantify the impact of the minority of super ties on countries’ scientific performance to provide new insights. These phases are described in more detail in the following subsections.

In this paper, we define collaborative tie strength *K*_*ij*_ as the cumulative number of publications coauthored by two countries, *i* and *j*, during the time period between their first and last publications. We calculate the average tie strength of each country *i* as follows, and refer to country *i* as the focused country:

$$\langle K_{i}\rangle =S_{i}^{-1}\Sigma_{j=1}^{s_{i}}K_{ij}$$ (1)

Average tie strength is calculated across the number of distinct collaborative countries *S*_*i*_ of focused country *i*. We use average collaborative tie strength 〈*K*_*i*_〉 to distinguish strong ties (*K*_*ij*_ ≥ 〈*K*_*i*_〉) from weak ties (*K*_*ij*_ < 〈*K*_*i*_〉). Super ties in the collaboration of each country are identified according to a tie strength threshold:

$$K_{i}^{c}=(\langle K_{i}\rangle -1)lnS_{i}$$ (2)

Each collaborative country *j* with *K*_*ij*_ > $K_{i}^{c}$ is labelled as a super tie of focused country *i* [36]. It should be emphasized that, as *S*_*i*_ represents the number of collaborative countries of a certain country, *S*_*i*_ is greater than or equal to 3 in our research. As this approach is centred around *i*, three types of tie definitions are not symmetrical; for example, if country *j* is a strong tie of *i*, then *i* is not necessarily a strong tie of *j*. According to [36], using an equivalent discrete exponential distribution of *P*(*K*_*ij*_), the super tie threshold $K_{i}^{c}$ is determined based on the extreme value criteria, which can be derived for any data following a discrete exponential distribution. In this research, we find that the statistical distribution *P*(*K*_*ij*_) of the international collaborative tie strength also follows the exponential distribution by exploring the distribution of normalized collaboration strength *x* = *K*_*ij*_/〈*K*_*i*_〉 using the Anderson-Darling test. Thus, the super tie threshold $K_{i}^{c}$ is adopted in the international collaboration relationship. The specific explanation of this threshold and the test procedure of exponential distribution are shown in S1 Appendix.

In this paper, among the three types of ties, strong ties are the main focus. Because almost all of the countries/regions analysed have more than one strong tie, we are able to quantify the added value of strong ties by using descriptive measures.

This paper adopts indicators of the stability and persistence of collaborative pairs of countries with strong ties. We use an explicit mathematical definition of international collaborative stability [20]: the consistent investment of a certain amount of effort into a relationship. Stability represents the degree of the long-term and similar investment made into a collaborative relationship. Within an arbitrary number of consecutive years, *N*, suppose that two countries, *i* and *j*, have a certain number of publications each year: $c_{ij}^{1},c_{ij}^{2}$, …, $c_{ij}^{N}$($c_{ij}^{1}$, $c_{ij}^{2}$, …, $c_{ij}^{N}$ ≥ 0, $\Sigma_{y=1}^{N}c_{ij}^{y}\neq 0$, *y* = 1, 2, …, *N*). The stability of collaboration between countries *i* and *j*, *S*_*i*,*j*_, is thus represented using the equation below, where max(*c*_*ij*_) refers to the maximum number of coauthored publications in a year.


$$S_{i,j}=1-\frac{\sum_{y=1}^{N-1}\left|c_{ij}^{y+1}-c_{ij}^{y}\right|}{\left(N-1)\cdot [\max(c_{ij})+1\right]}$$ (3)

In addition, we apply the following formula to calculate the persistence of collaboration *D*_*i*,*j*_ between countries *i* and *j* [48]. Compared with the definition of stability, persistence represents the degree of uninterrupted and continuous collaborative relationships. Moreover, stability emphasizes a certain investment in a relationship, while persistence reflects an uninterrupted collaborative relationship. The persistence is formally defined as follows:

$$D_{i,j}=N-s_{ij}+\lambda v_{i,j}$$ (4)

Given a consecutive year *N*, *s*_*ij*_ is the number of skip years without collaboration, which refers to the number of years in which countries *i* and *j* have zero coauthored articles within those *N* years; *v*_*i*,*j*_ is the number of intervals without collaboration between countries *i* and *j* within *N* years, defined as the number of contiguous time periods in which the two countries have no joint publication; and *λ* (0 < *λ* < 1) is a parameter to fit the model, which presents the importance of *v*_*i*,*j*_ in the model. In this article, we refer to Bu’s article and set *λ* = 0.5. Note that a country pair can have only one persistence value.

The scientific success of a collaborative pair is measured by the average scientific success of all publications coauthored by that pair. Thus, we calculate the yearly average number of citations (YANC) received per article, which indicates the impact of coauthored articles. The YANC minimizes the bias towards older papers, which tend to accumulate more citations. We also calculate the proportion of coauthored articles that have received at least 10 citations (CAP10C), which is actually the number of coauthored articles that have received at least 10 citations divided by the number of coauthored articles written by two countries.

## 4. Results

### 4.1 Characteristics of international scientific research collaboration

#### 4.1.1 Analysis of international collaboration tie strength

We summarize the collaborative relationships of each country/region according to our definitions of weak, strong and super ties. Table 2 shows the close collaborative relationships (i.e., strong and super ties) of high-productivity countries. 〈*K*_*i*_〉 indicates the average tie strength of country *i* with all collaborative countries. Due to space restrictions, we only display the collaboration of five high-productivity countries, and only the top five countries with the most collaborative papers are included in the collaborative country’s column. The close collaboration ties of all high-productivity countries are documented in S1 Table in S1 Appendix.

**Table 2:** Collaboration by high-productivity countries/regions.

Country___*i* /Region___*i*	Collaborator	Publications	<*K*_*i*_>	Tie Type	Number of Strong Ties	Number of Super Ties
US	Mainland China	22229	1334.42	strong tie, super tie	15	2
	Canada	7848		strong tie, super tie		
	UK	6710		strong tie		
	India	5707		strong tie		
	South Korea	5375		strong tie		
Mainland China	US	22229	802.18	strong tie, super tie	11	4
	Canada	4922		strong tie, super tie		
	Australia	4554		strong tie, super tie		
	Singapore	3551		strong tie, super tie		
	UK	3409		strong tie		
UK	US	6710	421.63	strong tie, super tie	18	3
	Mainland China	3409		strong tie, super tie		
	Germany	2304		strong tie, super tie		
	Australia	1625		strong tie		
	Canada	1410		strong tie		
Germany	US	5253	364.72	strong tie, super tie	17	3
	UK	2304		strong tie, super tie		
	Austria	1602		strong tie, super tie		
	Switzerland	1555		strong tie		
	France	1423		strong tie		
Canada	USA	7848	373.35	strong tie, super tie	14	2
	Mainland China	4922		strong tie, super tie		
	UK	1410		strong tie		
	Germany	1080		strong tie		
	France	974		strong tie		

Scientific research collaboration occurs between any two high-productivity countries. For these countries, most of the coauthored papers are written in collaboration with other high-productivity countries. The US is a strong tie of every other high-productivity country, indicating that collaboration with the US plays an important role in international collaboration for these countries. The US has an enormous number of publications and collaborative relations, and thus, it has advantages and power in international scientific research collaboration. Mainland China and France have the largest number of super ties, with 4 each. The UK has the largest number of strong ties, with 18.

Similarly, Table 3 shows the strong and super ties of low-productivity countries. Due to space limitation, we show only the five most productive countries in terms of the number of publications: Japan, South Korea, India, Singapore, and Italy. The collaboration of all low-productivity countries is presented in S2 Table in S1 Appendix. Table 3 and S2 Table in S1 Appendix show that the strong ties of every low-productivity country include one or more than high-productivity countries. The US is a strong tie of every low-productivity country except for Cuba. For both low- and high-productivity countries, international scientific research collaboration is based on national interests and aimed at enhancing countries/regions’ overall national strength and international competitiveness [49].

**Table 3:** Collaboration by low-productivity countries.

Country___*i* /Region___*i*	Collaborator	Publications	*<K*_*i*_ *>*	Tie Type	Number of Strong Ties	Number of Super Ties
Japan	USA	3435	204.77	strong tie, super tie	13	2
	Mainland China	3105		strong tie, super tie		
	South Korea	863		strong tie		
	Canada	779		strong tie		
	UK	753		strong tie		
South Korea	USA	5375	177.47	strong tie, super tie	12	3
	Mainland China	1254		strong tie, super tie		
	Japan	863		strong tie, super tie		
	Canada	571		strong tie		
	UK	352		strong tie		
India	USA	5707	173.59	strong tie, super tie	10	1
	Canada	777		strong tie		
	Mainland China	548		strong tie		
	Germany	537		strong tie		
	UK	535		strong tie		
Singapore	Mainland China	3551	194.35	strong tie, super tie	12	3
	USA	2695		strong tie, super tie		
	Australia	905		strong tie, super tie		
	Canada	610		strong tie		
	Hong Kong	575		strong tie		
Italy	USA	2407	167.16	strong tie, super tie	14	4
	Germany	1144		strong tie, super tie		
	France	1139		strong tie, super tie		
	UK	1002		strong tie, super tie		
	Spain	622		strong tie		

#### 4.1.2 Tie strength distribution

**Fig 4:**
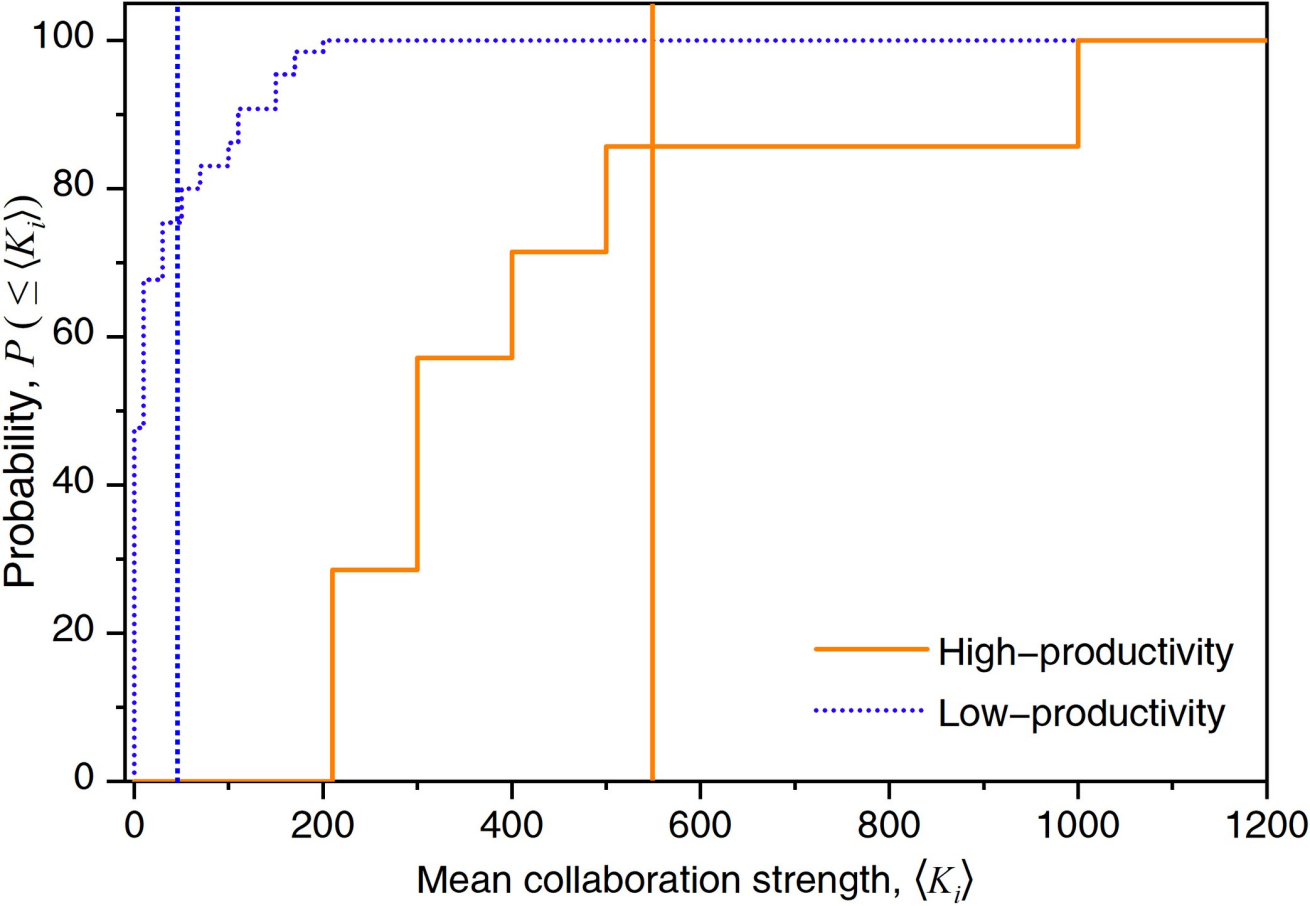
Cumulative distribution of mean collaboration strength 〈*K*_*i*_〉.

Here, we focus on the cross-sectional distribution of tie strength within the ego network. Fig 4 shows the cumulative distribution *P*(≤ 〈*K*_*i*_〉) of the mean tie strength 〈*K*_*i*_〉 of high- and low-productivity countries, which can vary over a wide range depending on a country/region’s involvement in international science activities. This measure captures the variability in collaboration strength. In Fig 4, high-productivity countries exhibit larger 〈*K*_*i*_〉 values. Vertical lines indicate the mean values. The figure shows that the average collaborative tie strength of high-productivity countries (with mean value 〈*K*_*i*_〉 ≈ 547.57) is generally higher than that of low-productivity countries (with mean value 〈*K*_*i*_〉 ≈ 47.17).

### 4.2 Impact of strong ties on international research performance

#### 4.2.1 Prevalence and impact of strong ties

**Fig 5:**
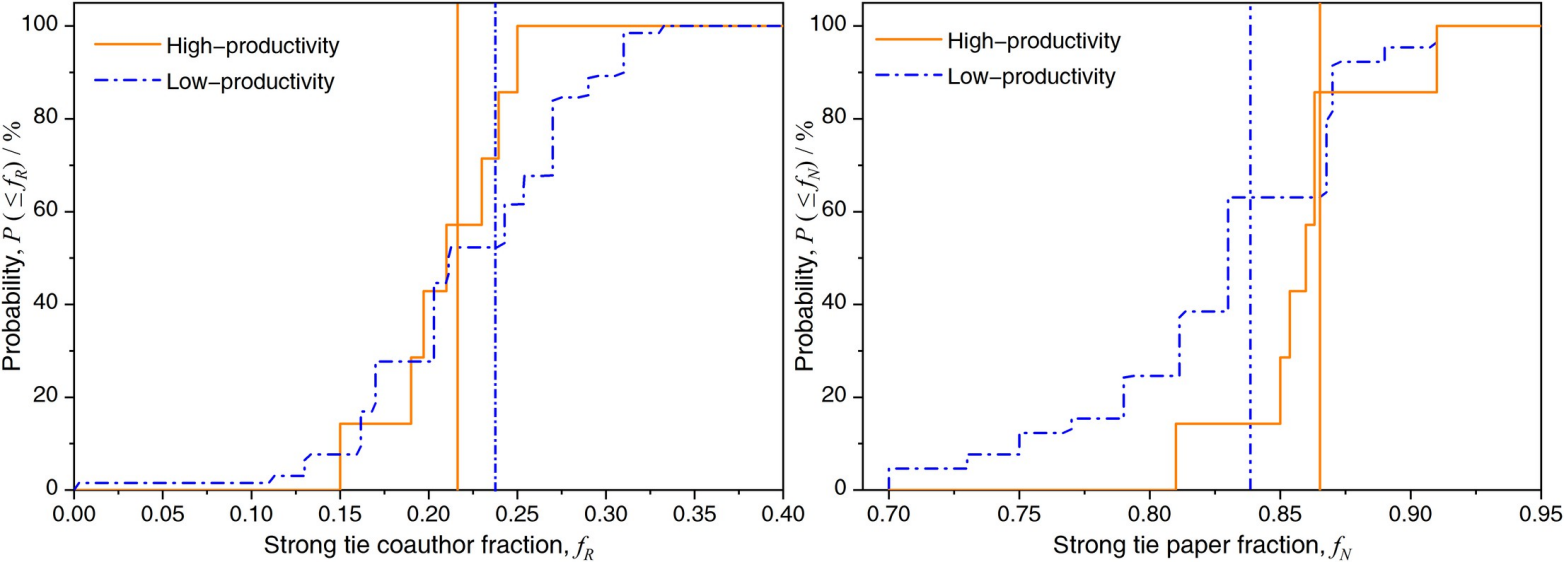
Frequency of strong ties: Vertical lines indicate the distribution mean. (a) Cumulative distribution of the fraction *f*_*R*,*i*_ of *S*_*i*_ coauthors that are strong ties. (b) Cumulative distribution of the fraction *f*_*N*,*i*_ of publications that include at least one strong tie.

How common are strong ties in international collaboration? We denote the number of co-author countries/regions that are strong ties as *S*_*R*,*i*_. The strong tie coauthor fraction *f*_*R*,*i*_ = *S*_*R*,*i*_/*S*_*i*_ measures the strong tie frequency on a per-collaborator basis, with a mean value of 〈*f*_*R*_〉 ≈ 0.236, which indicates that strong ties occur at an average rate of 1 in 4 coauthor countries/regions. Furthermore, Fig 5A shows the distribution *P*(≤*f*_*R*_) of two groups of countries. The mean value of the strong-tie coauthor fraction of high-productivity countries (with mean value 〈*f*_*R*_〉 ≈0.216) is generally smaller than that of low-productivity countries (with mean value 〈*f*_*R*_〉 ≈0.238). High-productivity countries have greater scientific capacity to attract more countries/regions as collaborators, so the proportion of strong ties in these countries is relatively small.

Fig 5B shows, on a per-paper basis, the fraction of a country/region’s publications coauthored with at least one strong tie, *f*_*N*,*i*_ (with mean value 〈*f*_*N*_〉 ≈ 0.844), with a mean of 0.866 for high-productivity countries and 0.842 for low-productivity countries. The establishment of close partnerships can reduce the risk of inefficient collaboration for countries/regions. Overall, the mean value of *f*_*N*,*i*_ for low-productivity countries is lower than that of high-productivity countries, indicating that high-productivity countries have higher levels of strong tie dependency than do their low-productivity counterparts.

In this subsection, we find that on average, a country/region’s publications coauthored with at least one strong tie account for more than 80% of all collaborative papers. Does collaboration with strong ties bring about benefits to countries/regions in terms of scientific research performance? In next subsection, we investigate the role of strong ties at the micro-level by analysing annual productivity and the citation impact of international publications.

#### 4.2.2 Impact of strong ties on annual productivity

We analyse the profile of collaborative research from 1993 to 2013, separating the data into nonoverlapping Δ*t*-year periods. Panel data for 72 countries/regions from 1993 to 2013 as well as two time periods, each lasting around 10 years, from 1993 to 2003 and 2004 to 2013, are used to build the regression model.

For country/region *i*, we label each strong-tie *j* with the indicator variable $R_{i}^{j}$≡1. The rest of the ties have the indicator variable $R_{i}^{j}$ ≡ 0. We then model the dependent variable *n*_*i*,*t*_/〈*n*_*i*_〉, which is productivity aggregated over Δ*t*-year periods, normalized by the baseline average calculated over the period of analysis. To better understand the factors contributing to productivity growth, in our regression model, we use four explanatory variables that are specific to country/region (*i*).

First, we calculate the average number of countries per publication ${\bar{c}}_{i,t}$ and the average number of authors per publication ${\bar{a}}_{i,t}$, using two proxies to represent labour input, coordination costs from the perspective of international collaboration and author collaboration. In addition, we calculate *N*_*i*,*t*_, the total number of publications up to year *t*, which is a measure of the focused country/region’s activeness and research profile. Finally, for each period *t*, we calculate the contribution of strong-tie collaborators *ρ*_*i*,*t*_, normalized by the contribution of all other collaborators to account for the possibility that the relative contribution of strong ties may affect productivity.


$$\rho_{i,t}=\frac{\sum_{j\left|R_{i}^{j}=1\right.}\Delta K_{ij}(t)}{\sum_{j\left|R_{i}^{j}\right.}\Delta K_{ij}(t)}$$ (5)

We include countries/regions in this analysis only if there are data of 5 years or more of international collaboration for which the denominator of *ρ*_*i*,*t*_ is nonzero. To deal with the nonstationarity of the data, we use the logarithmic forms of three variables. Variance inflation factors (VIF) are used in this study to test for multicollinearity. We adopt the criterion that the presence of multicollinearity is indicated by VIF values larger than 10. The results show that the VIF values of the independent variables are less than 3, indicating that there is no threat of multicollinearity problem [50]. The fixed and random effects models are two basic models for panel data analysis [51]. We employ the Hausman test to choose between fixed and random effects estimation for each regression performed, and the null hypothesis that the random effects model gives a consistent and efficient estimate is rejected (*p* < 0.01). Thus, the analysis is mainly based on fixed effects regression.

We implement the fixed effects model as follows:

$$\frac{n_{i,t}}{\langle n_{i}\rangle }=\beta_{i,0}+\beta \bar{{}_{c}}ln{\bar{c}}_{i,t}+\beta_{\bar{a}}ln{\bar{a}}_{i,t}+\beta_{N}lnN_{i,t}+\beta_{\rho }\rho_{i,t}+\epsilon_{i,t}$$ (6)

which accounts for country-specific time-invariant features (*β*_*i*,0_) with robust standard errors (SEs) to account for autocorrelation within each *i*. Table 4 shows the results of our model estimates for Δ*t* = 1 year. In addition to individual regressions for high- and low-productivity country groups, we run the regression for the full dataset (“All”).

**Table 4:** Parameter estimates for the productivity model of strong ties for *n*_i,*t*_/〈*n*_*i*_〉.

Dataset	A	*ρ*_*i*,*t*_	* $ln\bar{c_{l,t}}$ *	* $ln\bar{a_{l,t}}$ *	*lnN*_*i*,*t*_	*N*_*obs*._	*Adj*.*R*^*2*^
All	72	0.005	-0.896	0.906	0.478	1179	0.36
*p-value*		**0.033**	**0.009**	**0.000**	**0.000**		
High-productivity	7	0.003	-3.453	3.401	0.300	130	0.25
*p-value*		0.897	0.303	**0.018**	**0.000**		
Low-productivity	65	0.006	-0.891	0.833	0.492	1049	0.36
*p-value*		**0.027**	**0.011**	**0.000**	**0.000**		

Notes: Values significant at the *p*≤0.05 level are indicated in boldface. “All” indicates the combination of all datasets.

We observe a positive coefficient, *β*_*ρ*_ = 0.005 (*p*<0.05 for all datasets), meaning that larger contributions from strong ties are associated with above-average productivity and that strong ties play an important role in sustaining productivity growth. For example, let us consider the scenario where the average *ρ*_*i*,*t*_ value observed is 7.378. Then, let us consider a second scenario with *ρ*_*i*,*t*_ = 1, corresponding to equal input from strong ties and their counterparts. If all other parameters contribute a baseline productivity value of 1, then the additional contribution from *β*_*ρ*_ corresponds to a 7.378 *β*_*ρ*_/(1+7.378*β*_*ρ*_) = 4.2% productivity increase. We also find that above-average productivity is associated with a higher total number of publications of the focused country/region (*β*_*N*_>0, *p*<0.01 for all datasets). The coefficient of $ln\bar{a_{l,t}}$ is positive and significant in the three datasets, indicating that the larger the number of co-authors per paper, the more positive the effect is on the scientific research output of countries/regions. It’s interesting to note that in all three datasets, the coefficient of $ln\bar{c_{l,t}}$ is negative and significant, which suggests that more cooperative countries/regions may not necessarily increase production. For the regression analysis on high- and low-productivity countries, the *β*_*ρ*_ coefficient of high-productivity countries is 0.003, while that of low-productivity countries is 0.006. Moreover, strong ties contribute a 2.2% increase in scientific research productivity for high-productivity countries and a 4.5% increase for low-productivity countries. By observing the coefficient *β*_*ρ*_, we find that in international collaboration, collaboration with strong ties increases productivity more for low-productivity countries than for high-productivity ones.

Throughout different time intervals, strong ties also contribute to productivity advantages for countries/regions. A 2.8% increase in the productivity of a country/region’s publications is related to the effects of strong ties between 1993 and 2003, as shown in S3 Table in S1 Appendix; between 2004 and 2013, as shown in S4 Table in S1 Appendix, the productivity increased by 11.9% as a result of the benefits of strong ties.

#### 4.2.3 Impact of strong ties on the number of citations of international publications

It is very difficult to measure the impact of strong ties on the number of citations of a country/region’s publications, because old publications have more time to accrue citations than do new papers, so a direct comparison of raw citation counts for publications from different years is technically flawed. Therefore, we calculate the YANC, which indicates the impact of coauthored articles and minimizes the bias towards older papers. Then, we model the dependent variable *c*_*i*,*t*_, which is the average YANC aggregated over Δ*t*-year periods.

Following the previous step, we use the same explanatory variables as in Eq 6, which include the average number of collaborating countries per publication ${\bar{c}}_{i,t}$, the average number of co-authors per publication ${\bar{a}}_{i,t}$, the total number of publications *N*_*i*,*t*_ up to year *t*, and the normalized contribution of strong-tie collaborators *ρ*_*i*,*t*_. For the panel data regression method, we also use the Hausmann test to identify the appropriate regression model. The *p*-value shows that the fixed effects model is more suitable than is the random effects model for quantifying the impact of strong ties on the number of citations of publications. Thus, we implement fixed effects regression to estimate the parameters of the citation impact model.


$$c_{i,t}=\beta_{i,0}+\beta_{\bar{c}}ln{\bar{c}}_{i,t}+\beta_{\bar{a}}ln{\bar{a}}_{i,t}+\beta_{N}lnN_{i,t}+\beta_{\rho }\rho_{i,t}+\epsilon_{i,t}$$ (7)

Table 5 lists the (standardized) parameter estimates for Δ*t* = 1 year.

**Table 5:** Parameter estimates for the citation model of strong ties for *c*_i,t_ with Δ*t* = 1 year.

Dataset	A	*ρ*_*i*,*t*_	* $ln\bar{c_{l,t}}$ *	* $ln\bar{a_{l,t}}$ *	*lnN*_*i*,*t*_	*N*_*obs*._	*Adj*.*R*^*2*^
All	72	0.029	2.643	-1.445	-0.272	1108	0.24
*p-value*		**0.005**	**0.007**	0.071	**0.000**		
High-productivity	7	0.053	1.211	-9.677	0.514	139	0.33
*p-value*		0.095	0.848	**0.000**	**0.002**		
Low-productivity	65	0.030	2.704	-1.212	-0.310	969	0.28
*p-value*		**0.008**	0.090	0.158	**0.000**		

Notes: Values significant at the *p*≤0.05 level are indicated in boldface. “All” indicates the combination of all datasets.

We estimate *β*_*ρ*_ = 0.029 (*p*≤0.05 in the “All” dataset), indicating that a 18.8% increase in the number of citations of a country/region’s publications is related to the effects of strong ties. We argue that the reason for this may be that strong ties contribute to publication visibility. We also observe a positive coefficient, $\beta_{\bar{c}}$ = 2.643 (*p*<0.05 in the “All” dataset), of the number of collaborative countries parameter, meaning that average annual number of citations is also associated with the average number of collaborative countries of the focused country/region. Strong ties also result in citation gains for countries/regions for various time periods, as demonstrated in S5 and S6 Tables in S1 Appendix. Strong ties contributed to a 22.9% rise in citations between 1993 and 2003. A 17.3% increase in citations between 2004 and 2013.

For the regression results for high- and low-productivity countries, we can see that high-productivity countries (*β*_*ρ*_ = 0.053) have a *β*_*ρ*_ parameter that is higher than that of low-productivity countries (*β*_*ρ*_ = 0.030). In other words, the influence of strong ties on the number of citations of high-productivity countries increases by 28.8%, while that on the number of citations of low-productivity countries increases by 19.2%. This indicates that academic collaboration with strong ties provides more visibility and influence to high-productivity countries than to low-productivity countries.

Based on the above results, for high-productivity countries, strong ties provide more improvements to the number of citations of publications than to research productivity. That is, strong partners boost the citations of scientific research achievements more for high-productivity countries; in contrast, strong ties boost the quantity of scientific research achievements more for low-productivity countries.

### 4.3 Persistence and stability of international collaboration with strong ties

In this section, we measure and analyse the persistence and stability of collaboration within country pairs with strong ties to analyse which types of international strong ties tend to have a high citation impact. In a collaborative pair, if country/region *i* is a strong tie of *j* or if both *i* and *j* are strong ties of each other, then in this subsection, we regard it as a pair with a strong tie. In the following analysis, the collaborative pairs mentioned all refer to collaborative country pairs with strong ties. The characteristics of stability and persistence in two different time periods—1993–2003 and 2004–2013—are also taken into consideration, in addition to exploring the stability and persistence of international collaboration utilizing publications from 1993 to 2013.

#### 4.3.1 Persistence of international scientific collaboration

Perseverance has long been considered a hallmark of academic success [52]. An author’s persistent effort, represented as a persistent presence in academic publishing, has been found to be associated with high influence and success in his/her academic career [53]. However, little attention has been paid to how persistence in maintaining international collaborative relationships affects a country’s research performance. Referring to the measurement of persistence, we quantify and analyse the persistence of international collaboration.

To measure the scientific performance of a collaborative pair, we calculate the YANC received per paper and the CAP10C for collaborative pairs.

**Fig 6:**
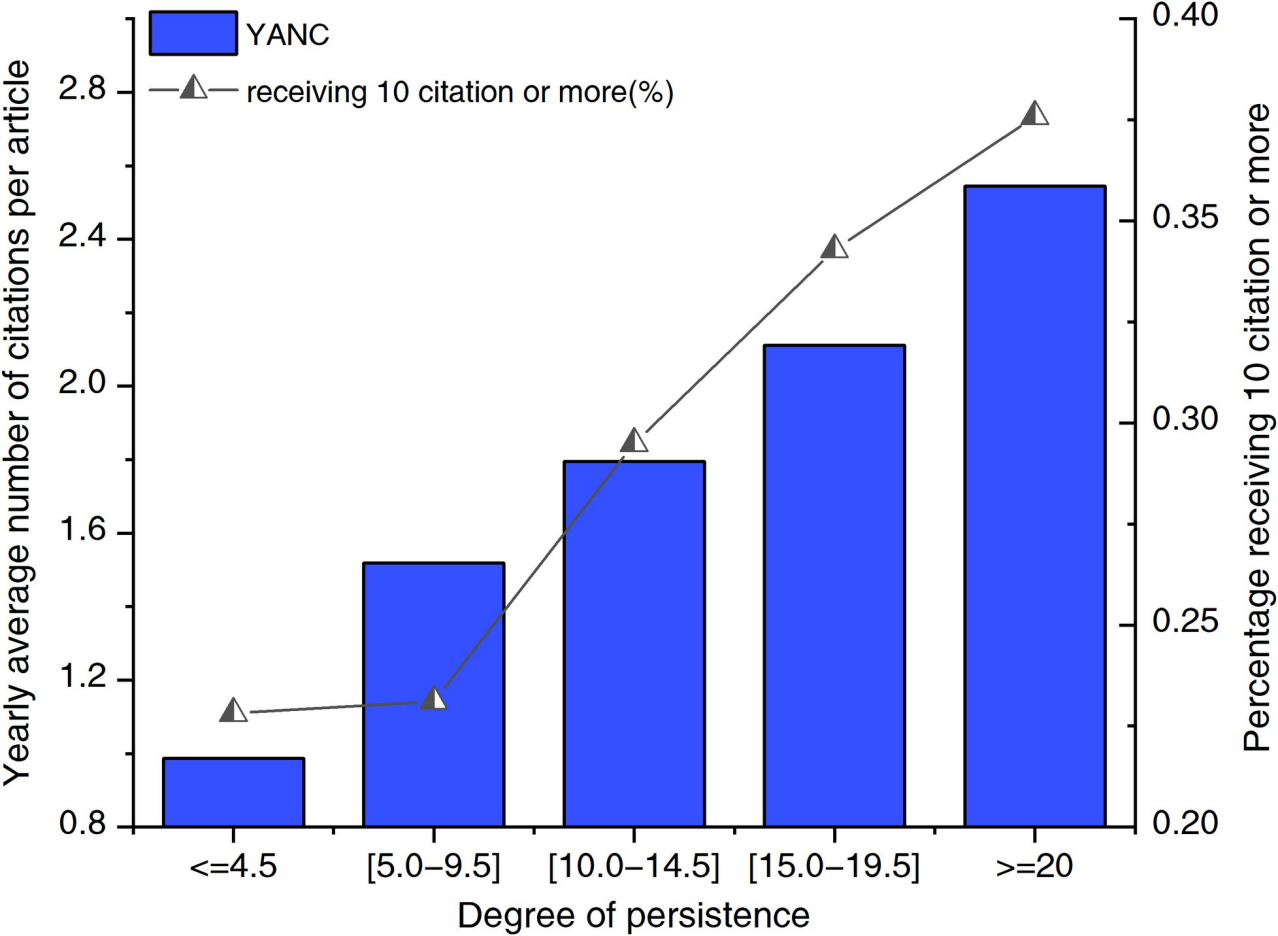
Yearly average number of citations (YANC) received per article and the proportion of coauthored articles that have received at least 10 citations (CAP10C) per year for different persistence groups in 1993–2013.

According to the value of persistence, we divide the collaborative country pairs into five groups. We label the collaborative pairs with persistence (0, 4.5] as low-persistence pairs, [5.0, 9.5] as low-to-medium-persistence pairs, [10.0, 14.5] as medium-persistence pairs, [15.0, 19.5] as medium-to-high-persistence pairs and > = 20 as high-persistence pairs. Fig 6 shows the YANC and CAP10C values of the different groups. We can see that groups with a generally higher degree of persistence have higher YANC and CAP10C indicators and that the high group (pairs with a persistence measure greater than 40) has the highest YANC and CAP10C indicators. Therefore, the YANC and CAP10C are shown to increase with the increase in the degree of persistence. Additionally, we look at the connection between persistence and scientific performance in different time intervals. The similar pattern can be observed in different time intervals—YANC and CAP10C increased with persistent elevation—as illustrated in S6 and S7 Figs in S1 Appendix.

The results emphasize the importance of maintaining continuous international scientific collaboration. Collaborators with a lower level of persistence should take time and effort to understand each other’s research status, and collaborators with a high level of persistence should work tirelessly to optimize their research process based on their long-term persistent collaborations. In academic research, the high degree of familiarity of high-persistence groups helps them better utilize and share resources with each other. Similarly, in medical research and other “cumulative sciences”, where the cumulative production of information is mandatory [52], persistence is expected to accrue more research resources and achieve career success.

#### 4.3.2 Stability of international scientific collaboration

As science has gradually become a team activity, it has become important to understand the nuances of team stability [2]. However, there are few studies examining the role of collaborative stability in international scientific research. In this regard, we aim to draw on the definition of stability [20] to quantify the stability of international collaboration.

**Fig 7:**
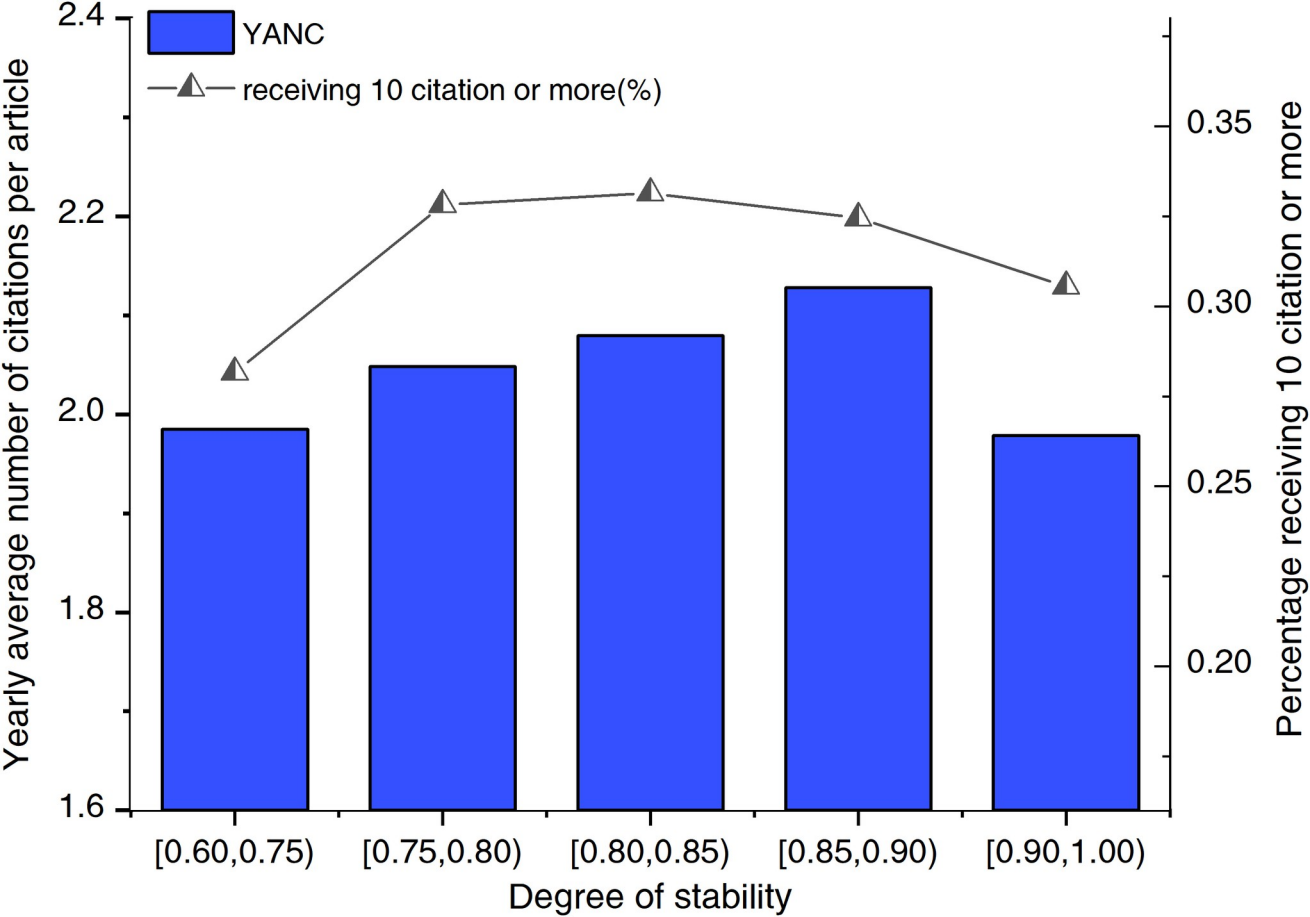
Yearly average number of citations (YANC) received and proportion of coauthored articles that have received at least 10 citations (CAP10C) per year for different stability groups in 1993–2013.

Based on the stability value, we divide collaborative country pairs into five groups. We call the collaborative pairs with stability [0.60, 0.75) as low-stability pairs, those with [0.75, 0.80) as low-to-medium-stability pairs, those with [0.80, 0.85) as medium-stability pairs, those with [0.85, 0.90) as medium-to-high-stability pairs and those with [0.90, 1.00) as high-stability pairs. Fig 7 shows the relationship between the stability of collaborations and their success, measured as the YANC and CAP10C, grouped into five bins based on their stability measures.

Generally, groups with a high stability value can have more chances to increase their YANC and CAP10C values. However, as shown in Fig 7, those collaborators with a medium–high stability (collaborations with a degree of stability between 0.85 and 0.90) have the greatest YANC, which is even greater than that of the most stable group. CAP10C is the highest for the medium stable pairs, and decreases in the medium–high stable group and the most stable group.

We also explore the relationship between stability of collaboration and scientific performance between countries/regions in different time intervals. The same trend as that during the entire era of collaboration is observed in 2004–2013, as shown in S9 Fig in S1 Appendix: Collaborations with a medium–high level of stability have the highest YANC. S8 Fig in S1 Appendix, shows the relationship between stability of collaboration and scientific performance in 1993–2003. It shows that groups with a high stability value can have more chances to increase their YANC and CAP10C values. The high-stability group has the highest YANC and CAP10C values.

Stability represents the degree of the long-term and consistent investment into a collaborative relationship. Country pairs with a higher degree of stability have better research performance than those with lower stability. Stability may be important for fostering knowledge exchange among participating countries and producing achievements of a high calibre [52, 54]. Access to shared information and resources and improved team cohesiveness may all contribute to the performance of collaborative partnerships that retain a high degree of stability. However, the relationship between constant investment and the improvement in scientific output and effect is not strictly linear, and completely stable teams may suffer poor returns due to excessive embeddedness [20].

#### 4.3.3 Relationships among persistence, stability and research performance

**Fig 8:**
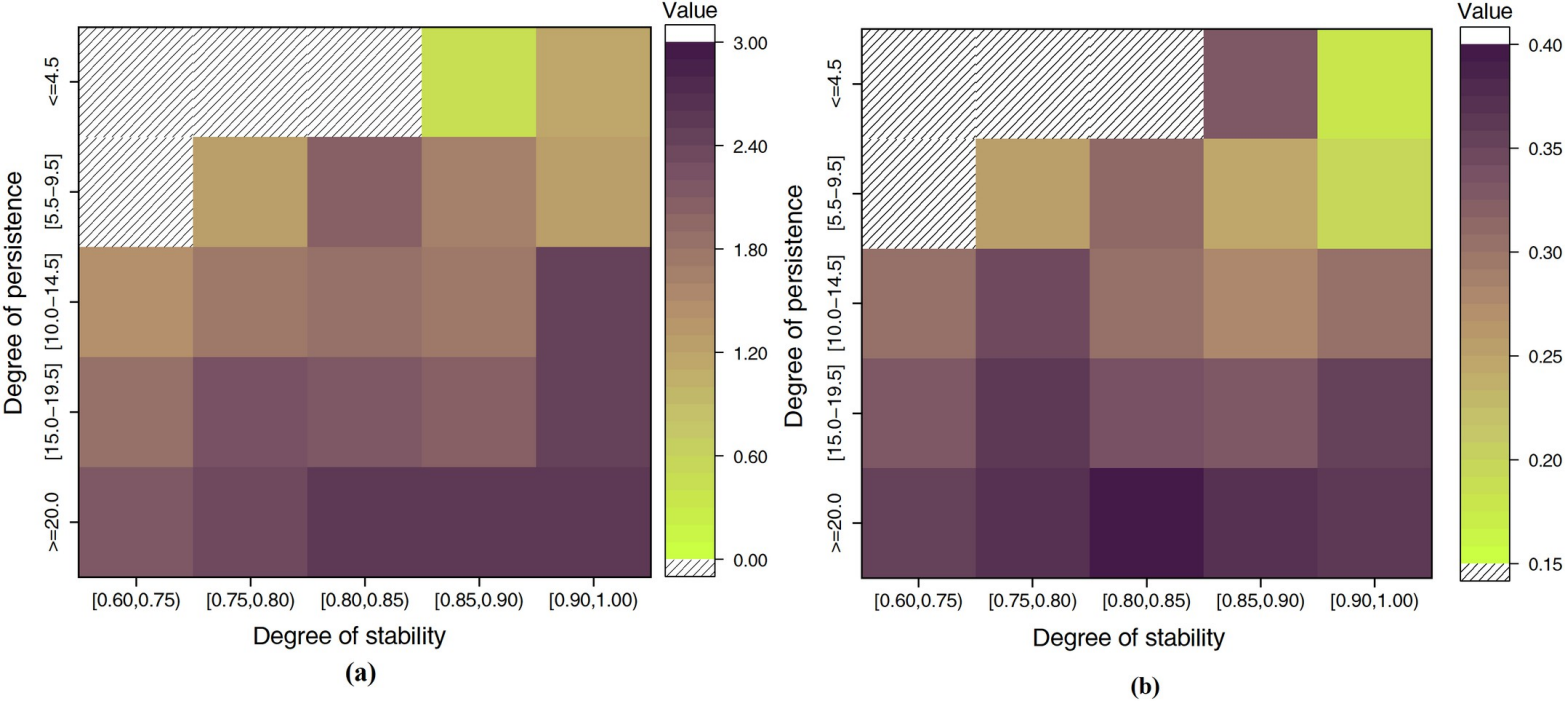
Relationships among collaboration persistence, stability and research performance from different aspects in 1993–2013. (a) the yearly average number of citations (YANC) received and (b) the proportion of coauthored articles that have received at least 10 citations (CAP10C); shading is proportional to the value of the research performance indicators of the collaborative country pairs with the corresponding degree of stability and persistence.

To better understand international scientific collaboration, we need a host of perspectives. Both persistence and stability measure the characteristics of collaboration counts of a given country pair. To understand how persistence may interact with stability to affect the citation impact of international publications is therefore of interest. Thus, here, we use collaborative persistence and stability to better understand the structure of successful international scientific collaboration. In Fig 8, we show two heatmaps, each with persistence and stability grouped into five groups. The horizontal axis represents the degree of persistence of country pairs, and the vertical axis represents the degree of stability of country pairs. Cells in different heatmaps represent different research performance indicators: the YANC or the CAP10C.

Fig 8A shows the relationships among persistence, stability and the YANC in the period from 1993 to 2013. Each cell represents the YANC of collaborative country pairs with the combination of the stability and persistence groups. Those country pairs that have a high degree of persistence (> = 20) along with a medium degree of stability [0.80, 0.85) exhibit the best research performance. In addition, Fig 8A indicates that for collaboration pairs with different levels of stability, the group with a high degree of persistence tends to have a high YANC, while pairs with moderate stability have better performance. Fig 8B shows the CAP10C of a collaborative pair and the way in which it relates to stability and persistence. We see that articles with the highest impact are written by collaborative pairs with high persistence (> = 20) and medium stability [0.80, 0.85). Low-persistence pairs are found to have a lower average CAP10C value than high-persistence pairs, and this result is similar to the result discussed in the last subsection.

The correlations between persistence, stability, and the YANC from 1993 to 2003 are depicted in S10a Fig in S1 Appendix. The highest research performance is shown in collaborations with high levels of stability (>0.85) and persistence (> = 10). It has been discovered that low-persistence couples have a lower average YANC value than high-persistence pairs. S10b Fig in S1 Appendix shows the CAP10C of a collaborative pair and the way in which it relates to stability and persistence. We see that collaborations with high persistence (> = 10) and medium stability (0.75, 0.80) produce the papers with the highest impact.

According to S11a Fig in S1 Appendix, the collaborations that have a high degree of stability (>0.85) and a medium-high degree of persistence [8.5, 9.5] during the period of 2004 to 2013 exhibit the highest research performance. Additionally, according to S11a Fig in S1 Appendix, the group with a high level of persistence typically has a high YANC for collaborations with varying levels of stability. In S11b Fig in S1 Appendix, publications with the highest impact are written by collaborative pairs with medium-high persistence [8.5, 9.5] and medium-high stability (0.80, 0.85].

### 4.4 Impact of super ties on international scientific research performance

Most of the abovementioned research on international collaborative relations has focused on strong ties between countries/regions. In previous sections, we find that strong ties contribute to above-average productivity and a higher number of citations per publication for countries/regions. In this subsection, for the minority of international collaborations characterized by super ties, we quantify the impact of these ties on countries/regions’ research performance to draw new insights.

#### 4.4.1 Impact of super ties on annual productivity

We implement a fixed effects regression model to determine the sign and strength of the contribution of super ties to countries/regions’ productivity. The dependent variable *n*_*i*,*t*_/〈*n*_*i*_〉 and three independent variables (${\bar{c}}_{i,t}$, ${\bar{a}}_{i,t}$ *N*_*i*,*t*_) in the model are the same as those in Eq 6. To quantify the contribution of super ties, we use an explanatory variable, *φ*_*i*,*t*_. For country/region *i*, we label each super tie *j* with indicator variable $U_{i}^{j}$≡1. The rest of the ties take the indicator value $U_{i}^{j}$≡0.


$$\varphi_{i,t}=\frac{\sum_{j\left|U_{i}^{j}=1\right.}\Delta K_{ij}(t)}{\sum_{j\left|U_{i}^{j}=0\right.}\Delta K_{ij}(t)}$$ (8)

The fixed effects regression model is as follows:

$$\frac{n_{i,t}}{\langle n_{i}\rangle }=\beta_{i,0}+\beta_{\bar{c}}ln{\bar{c}}_{i,t}+\beta_{\bar{a}}ln{\bar{a}}_{i,t}+\beta_{N}lnN_{i,t}+\beta_{\varphi }\varphi_{i,t}+\epsilon_{i,t}$$ (9)

In the regressions, we only include countries/regions with data of 5 years or more of international collaboration, where *φ*_*i*,*t*_ is nonzero. In Table 6, we present the results of the fixed effects regression for Δ*t* = 1 year.

**Table 6:** Parameter estimates for the productivity model of super ties for *n*_*i*,*t*_/〈*n*_*i*_〉.

Dataset	*A*	*ϕ*_*t*_	* $ln\bar{c_{l,t}}$ *	* $ln\bar{a_{l,t}}$ *	*lnN*_*i*,*t*_	*N*_*obs*._	*Adj*.*R*^*2*^
All	72	0.033	-0.781	0.657	0.528	1224	0.27
*p-value*		**0.027**	**0.012**	**0.000**	**0.000**		
High-productivity	7	0.003	-3.294	3.675	0.273	133	0.00
*p-value*		0.985	0.360	**0.021**	**0.000**		
Low-productivity	65	0.033	-0.767	0.603	0.536	1091	0.32
*p-value*		**0.030**	**0.015**	**0.001**	**0.000**		

Notes: Values significant at the p≤0.05 level are indicated in boldface. “All” indicates the combination of all datasets.

We observe a positive coefficient, *β*_*φ*_ = 0.033 (*p*≤0.05 for all datasets), meaning that larger contributions by super ties are associated with above-average productivity. Our calculations show that the contribution of super ties leads to a 4.5% increase in scientific research productivity for countries/regions, which is greater than the increase in productivity brought about by strong ties. As shown in S7 Table in S1 Appendix, strong ties help countries/regions’ scientific research productivity increase by 4% between 1993 and 2003. According to S8 Table in S1 Appendix, there is an 8.2% growth in productivity between 2004 and 2013.

In the model results, the *β*_*φ*_ coefficient of high-productivity countries is 0.003, while that of low-productivity countries is 0.033. Thus, super ties are found to contribute a 4.0% increase in scientific research productivity for high-productivity countries and a 4.5% increase for low-productivity countries.

#### 4.4.2 Impact of super ties on the number of citations of international publications

Referencing Eq 7, we implement fixed effects regression to quantify the impact of super ties on the number of citations. The explanatory variables include the average number of collaborative countries per publication ${\bar{c}}_{i,t}$, the average number of collaborating authors per publication ${\bar{a}}_{i,t}$, the total number of publications up to year *t*, *N*_*i*,*t*_, and the normalized contribution of super tie collaborators *φ*_*i*,*t*_.


$$c_{i,t}=\beta_{i,0}+\beta_{\bar{c}}ln{\bar{c}}_{i,t}+\beta_{\bar{a}}ln{\bar{a}}_{i,t}+\beta_{N}lnN_{i,t}+\beta_{\varphi }\varphi_{i,t}+\epsilon_{i,t}$$ (10)

Table 7 lists the parameter estimates for Δ*t* = 1 year.

**Table 7:** Parameter estimates for the citation model of super ties for *c*_*i*,*t*_ with Δ*t* = 1 year.

Dataset	*A*	*ϕ*_*t*_	* $ln\bar{c_{i,t}}$ *	* $ln\bar{a_{i,t}}$ *	*lnN*_*i*,*t*_	*N*_*obs*._	*Adj*.*R*^*2*^
All	72	0.182	1.533	0.022	-0.389	1220	0.20
*p-value*		**0.014**	0.303	0.980	**0.000**		
High-productivity	7	0.632	3.668	-10.855	0.511	141	0.10
*p-value*		**0.034**	0.558	**0.000**	**0.002**		
Low-productivity	65	0.180	1.538	0.229	-0.421	1079	0.25
*p-value*		**0.022**	0.330	0.806	**0.000**		

Notes: Values significant at the p≤0.05 level are indicated in boldface. “All” indicates the combination of all datasets.

As seen from Table 7, for the full dataset, the coefficient of the contribution of super ties *β*_*φ*_ is 0.182. In other words, a 21.4% increase in the number of citations of a country/region’s publications can be traced to the effects of super ties, which is much higher than the increase in the number of citations brought about by strong ties. Super ties, according to S9 Table in S1 Appendix, increase the number of citations in a country’s international publications by 31.1% between 1993 and 2003. According to S10 Table in S1 Appendix, super ties contributed a 14.9% growth of citations between 2004 and 2013.

In addition, the influence of super ties increases the number of citations of high-productivity countries by 43.3% and those of low-productivity countries by 21.5%. Similar to strong ties, super ties lead to more influence gains for the high-productivity countries and more productivity gains for the low-productivity countries.

## 5. Discussion

International collaboration plays an important role in the production and dissemination of new scientific knowledge. An increasing share of scientific papers is co-authored by scientists from two or more countries/regions, which results in the emergence of international collaboration network [55]. Previous studies have mainly emphasized on the structural and dynamic properties of the international collaboration networks [40], and the effects that international collaboration has on scientific performance [56]. Tie strength has been examined as an antecedent of creativity [57], whereas the effect of tie strength in the international collaboration network has been largely neglected. The strength of relationship (i.e., intensity of connection in collaboration network) is a determining factor for a country’s research performance [58]. Collaborative tie strength involves only two collaborators, which allows for the drawing of more direct management recommendations and policies. Thereupon, this study aims to investigate the relationship between international collaborative ties and scientific performances, with special emphasis on the effect of collaborative ties, as well as how they are sustained. The following conclusions can be drawn from the analysis of international publications of 72 countries/regions from 1993 to 2013.

To explore the collaborative relationships between countries/regions, we quantify tie strength using an egocentric perspective of the collaboration network. Our analysis indicates that more than 3/4 (i.e., approximately 0.77) of the international collaboration ties analysed are weak. However, the remaining strong ties represent collaboration investments that can indeed have significant long-term effects for countries/regions. Although a country/region’s strong ties account for a lower proportion (i.e., occur at an average rate of 1 in 4 collaborators) of collaborative relationships, countries/regions share on average 84% of articles with their strong-tie collaborators. Many scholars have proposed various explanations for why such extremely strong international collaboration exists [59]. The greater skill set and complementarity that such partnerships bring about can provide an international competitive advantage [60]. So, is there a measurable advantage for a country/region’s research performance associated with collaborating with strong ties? On the one hand, our results indicate that strong ties contribute to a 4.2% increase in scientific research productivity for countries/regions, which shows that strong ties play a crucial role in countries/regions’ sustained productivity growth. On the other hand, coauthoring with strong ties is associated with a 18.8% increase in countries/regions’ number of citations, which may arise from the extra visibility from the substantial reputations of strong ties.

Continuous and close scientific collaboration may contribute to the production of international papers, to investigate how these strong ties are sustained, the persistence and stability characteristics of international collaboration are analysed. Collaborators with a high degree of persistence tend to receive more citations, persistent collaboration and less interference help build strong trust between countries/regions and bring long-term academic success. This result is similar to previous research revealing that persistence is closely related to scientific success [61]. For stability, international collaborative relationships that maintain a moderate high level of stability may receive a benefit resulting from shared resources and knowledge [62]. By considering persistence and stability together, we gain a better understanding of the structure of international collaboration with strong ties. In international collaboration, it is important to maintain the existing close and consistent collaboration relationship. But on the other hand, due to high embeddedness, totally stable collaborations may not have the highest return. Thus, expanding the collaboration network and gaining access to new knowledge from other countries/regions are also good approaches.

Our quantitative results are in accordance with literature suggesting the general trend of international collaborative work on the national level [21]. Our measures of the collaborative tie strength reveal that the close collaborative relationships (i.e., strong ties) exist in the international collaboration network. Extant literature explores the driving mechanisms behind these collaborative ties’ formation, e.g., similarity [63] and connectivity [64] are mechanisms for collaboration formation in real world networks [65]. There is generally more international collaboration between countries with larger numbers of international papers. It supports the theoretical argument of ties being more likely established between countries with higher rankings in the quantity of international papers [66], indicating the existence of a “rich-get-richer” phenomenon. Meanwhile, countries with high international publication output have strong relationships with numerous countries. They attract many countries/regions to form tight alliances. This may lead to an increasing connectivity within the network that implies the potential for knowledge transfer [65]. On the other hand, the diversity of the research teams involved may explain why international papers are more highly cited than domestic papers [67]. One should realize that the diverse international alliance partnership also provides access to a broader extent of external knowledge.

The findings of our study provide several implications for practicing managers concerning the question on how to configure international alliance relationships to promote scientific research performance. Global teamwork has evident consequences for the efficiency of scientific activities [6], the choice to start or develop the collaboration with countries can be an important strategic consideration with long-term implications. Countries’ egocentric international collaboration networks have a mixture of weak and strong ties (including super ties), and network ties are heterogeneous [38]. Instead of simply studying the overall tie strength of the network, these complicated configuration characteristics should be studied. As we have quantitatively demonstrated, a persistent research partner can have a positive impact on the sustainability and growth of international scientific research, so how to establish and maintain strong-tie relationships are important to consider for policy makers. There is a need to understand the dynamics of strong ties in international collaborative research to formulate best practices for collaborative work in journal publication. In addition, since the influence of super tie on scientific research performance is even greater than that of strong tie, countries can choose to develop their strong ties. Countries and researchers should make decisions about how to invest their resources in collaboration, whether they are physical, such as equipment, or intangible, such as social capital, in a careful manner [68]. Overall, we suggest that strong international alliance partnerships can provide a better international collaboration environment for collaboration activities, and deep trust has a positive effect for both collaborative countries.

## 6. Conclusions

The main purpose of this study is to explore how the bonding of collaboration ties contributes to countries/regions’ international scientific performances. It enriches the study of collaboration ties between countries/regions from the view of egocentric international collaboration networks. In an international collaboration network, two countries/regions are connected if they have coauthored a paper, and all collaborations are divided into dyadic relationships, which is the most central part of the collaboration network [69]. Second, this work explores how the collaboration ties of different degrees of compactness affect the scientific research performance of countries/regions. Notably, the results confirm that the establishment of a strong tie relationship contributes to above-average productivity and citation frequency, thus identifying these partnerships as a significant factor in the scientific development of a country/region. Third, this work investigates how the stability and persistence of strong ties interact to influence the success of international articles.

This paper provides an exploratory study of the effect of strong ties in the international collaboration, but there are still some limitations. First, ArnetMiner is an effective dataset to explore the contribution of strong relationships in international collaboration, but it also had limits. For example, the data solely contains articles in the field of computer science; citations from journals in other fields are not taken into account. We will expand the dataset of papers in more disciplines to precisely elucidate countries’ international collaboration. Second, the strong collaborative relationship between two subjects can be extended to the larger group to further expand the scope of the study. Last, in future studies, we will further explore the characteristics and effects of super ties, thus enriching and extending the existing findings.

## Supporting information

S1 Appendix (DOCX)
