# Supplementary material for: Quantifying the impact of strong ties in international scientific research collaboration
Source: PLoS One. 2023 Jan 17;18(1):e0280521. doi: 10.1371/journal.pone.0280521 (PMC9844855; doi:10.1371/journal.pone.0280521)
Supplement: S1 Appendix — (DOCX) [file pone.0280521.s001.docx]

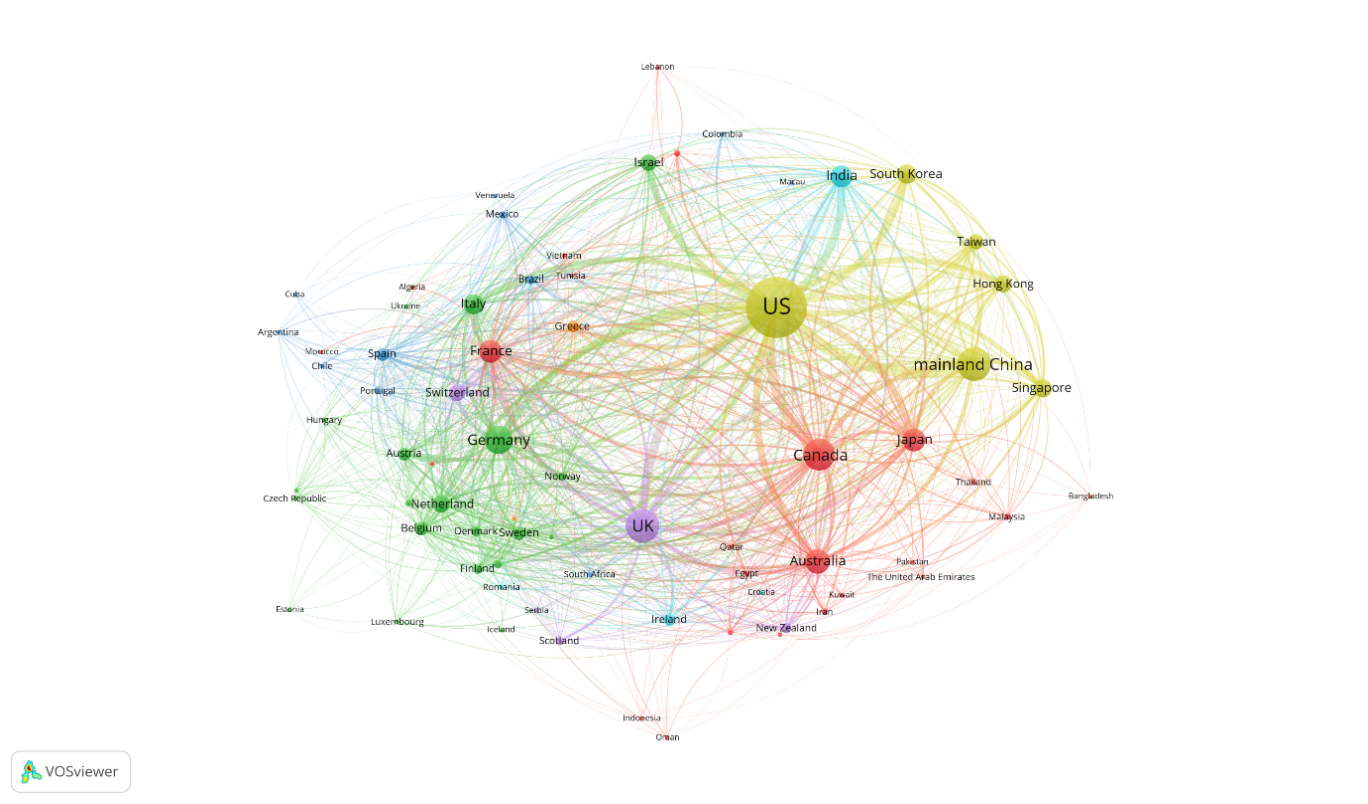


**S1 Fig. International scientific collaboration network in 1993-2003.**


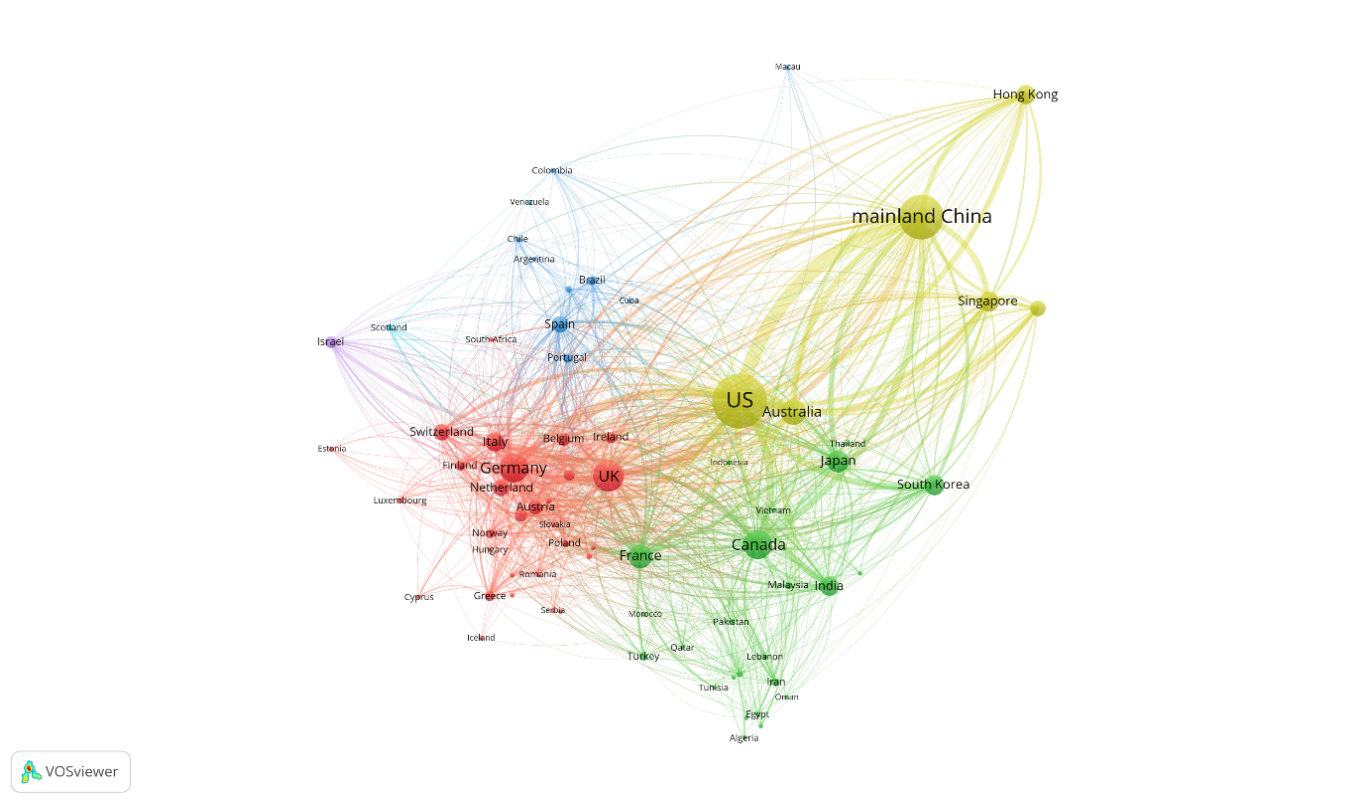


**S2 Fig. International scientific collaboration network in 2004-2013.**

The super tie threshold $K_{i}^{c}$ is applied in this research. The threshold $K_{i}^{c}$ can be derived for any data following a discrete exponential distribution [1]. To explore the applicability of this threshold and better understand the distribution of collaboration tie strength, we aggregated the tie strength data across all research profiles, using the normalized collaboration strength ${x=K}_{ij}/\left\langle K_{i} \right\rangle$.

We present the histogram of normalized tie strength in Fig.S3, which shows the characteristic of a left skewed distribution. We may infer from the blue density curve in the histogram that the data essentially follows an exponential distribution.


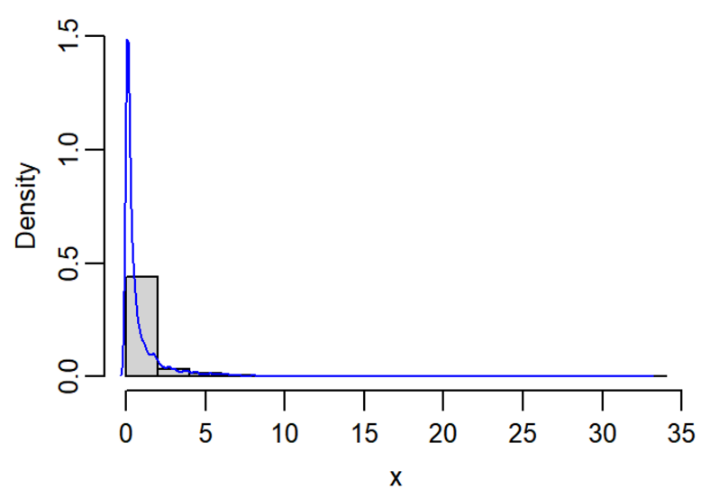


**S3 Fig. Histogram of collaboration normalized tie** **strength.**

The R package “fitdistrplus” [2] is used to assess the resemblance of the data distribution to exponential and other distributions. The data of normalized tie strength is represented as a blue dot in the Cullen and Frey graph (Fig.S4). The closer the point is to the distribution’s legend, the tie strength data is more likely to fit that distribution. Fig.S4 shows that the data distribution represented by the blue dot is more similar to the beta, exponential, gamma, and lognormal distributions. The beta distribution is abandoned since it is only appropriate for data sets in the range [0,1].


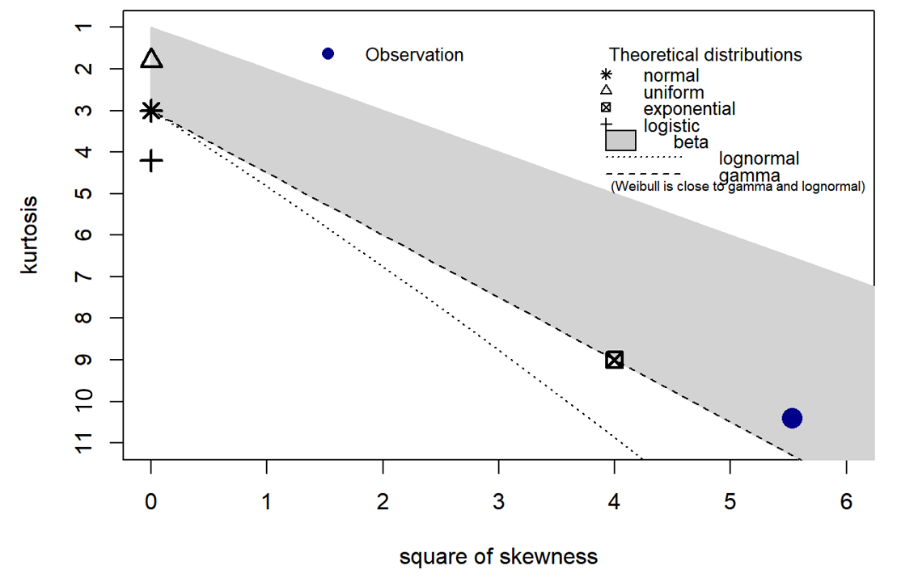


**S4 Fig. Cullen and Frey graph of collaboration normalized tie** **strength.**

As a result, we further examine the fitting degree of the normalized tie strength data to these three distributions. The following are the standard errors of fitting results between the normalized tie strength and various distributions: 0.015 for the exponential distribution, 0.017 for the gamma distribution, and 0.019 for the lognormal distribution. The exponential distribution’s standard error is the smallest, indicating that the data distribution is closer to the exponential distribution. The Anderson-Darling test (A-D test) is then applied on normalized tie strength data to assess if it follows an exponential distribution. The result shows that *p* value > 0.1, suggesting that the normalized tie strength follows an exponential distribution.

Then, we can assume that normalized tie strength ${x=K}_{ij}/\left\langle K_{i} \right\rangle$ follows the exponential distribution with parameter $\lambda$. Maximum Likelihood Estimation states that, if *x* follows an exponential distribution with parameter $\lambda$, then:

$$f\left( \lambda;x \right)=\left\{ \begin{aligned} \lambda e^{-\lambda x}, x>0 \\ 0 ,x\leq0 \end{aligned} \right.$$

The maximum likelihood estimates for parameter $\lambda=1/\left\langle x \right\rangle$. As a result, in this research, the estimator $\lambda=1$ with $\left\langle x \right\rangle=1$. That is, $f\left( x \right)=e^{-x} (x>0).$ We plot the cumulative distribution curve of normalized strength ${x=K}_{ij}/\left\langle K_{i} \right\rangle$ and the exponential distribution $e^{-x}$ in Fig.S5, the finding shows that the $P(\geq x)$ distribution is in good agreement with $e^{-x}$ distribution, demonstrating the suitability of this threshold.

The following is the specific explanation of this extreme value. According to [3], an estimate for $K_{i}^{c}$ is given by the extremal criterion that one of the $S_{i}$ observations of collaborators has a value that is greater than or equal to $K_{i}^{c}$, that is, $1/S_{i}=\sum_{K_{ij}>K_{i}^{c}}^{\infty} P(K_{ij})$. We discover, using the Maximum Likelihood Estimation, that the distribution of the $P(\geq x)$ follows the exponential distribution $e^{-x}$, i.e., $P(K_{ij})\propto exp(-k_{i}K_{ij})$. It denotes that $1/S_{i}=exp(-k_{i}K_{ij})$, with the analytic relation $\left\langle K_{i} \right\rangle=\sum_{K_{ij}=1}^{\infty} K_{ij}P(K_{ij})=\frac{e^{k_{i}}}{e^{k_{i}}-1}\approx1+1/k_{i}$. By combining the above equations, we can obtain the super tie threshold $K_{i}^{c}=\left( \left\langle K_{i} \right\rangle-1 \right)lnS_{i}$. It should be emphasized that, as $S_{i}$represents the number of collaborative countries of a certain country, $S_{i}$ is greater than or equal to 3 in our research.


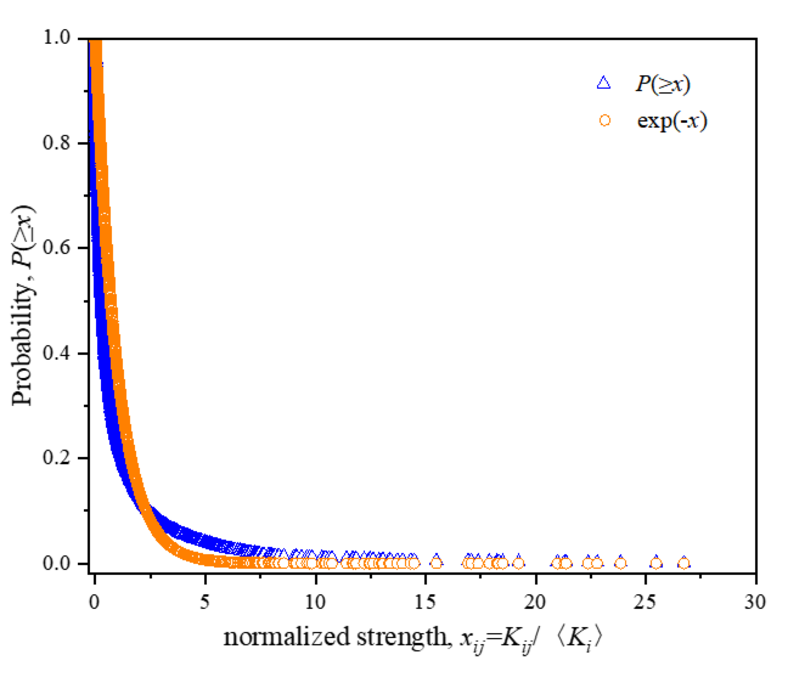


**S5 Fig. Characteristic measures of collaboration normalized tie** **strength.**

**S1 Table. Collaboration by high-productivity countries/regions.**

| **Country___*i* /Region___*i*** | **Collaborator** | **Publications** | **<*K_i_*>** | ***Kc*** | **Tie Type** |
| --- | --- | --- | --- | --- | --- |
| USA | Mainland China | 22229 | 1334.42 | 5683.95 | strong tie,super tie |
| USA | Canada | 7848 | 1334.42 | 5683.95 | strong tie,super tie |
| USA | UK | 6710 | 1334.42 | 5683.95 | strong tie,super tie |
| USA | India | 5707 | 1334.42 | 5683.95 | strong tie |
| USA | South Korea | 5375 | 1334.42 | 5683.95 | strong tie |
| USA | Germany | 5253 | 1334.42 | 5683.95 | strong tie |
| USA | Australia | 4076 | 1334.42 | 5683.95 | strong tie |
| USA | Hong Kong | 3637 | 1334.42 | 5683.95 | strong tie |
| USA | Japan | 3435 | 1334.42 | 5683.95 | strong tie |
| USA | Taiwan | 2962 | 1334.42 | 5683.95 | strong tie |
| USA | Israel | 2941 | 1334.42 | 5683.95 | strong tie |
| USA | France | 2749 | 1334.42 | 5683.95 | strong tie |
| USA | Singapore | 2695 | 1334.42 | 5683.95 | strong tie |
| USA | Italy | 2407 | 1334.42 | 5683.95 | strong tie |
| USA | Switzerland | 1611 | 1334.42 | 5683.95 | strong tie |
| Mainland China | USA | 22229 | 802.18 | 3380.57 | strong tie,super tie |
| Mainland China | Canada | 4922 | 802.18 | 3380.57 | strong tie,super tie |
| Mainland China | Australia | 4554 | 802.18 | 3380.57 | strong tie,super tie |
| Mainland China | Singapore | 3551 | 802.18 | 3380.57 | strong tie,super tie |
| Mainland China | UK | 3409 | 802.18 | 3380.57 | strong tie,super tie |
| Mainland China | Japan | 3105 | 802.18 | 3380.57 | strong tie |
| Mainland China | Hong Kong | 2591 | 802.18 | 3380.57 | strong tie |
| Mainland China | Germany | 1340 | 802.18 | 3380.57 | strong tie |
| Mainland China | South Korea | 1254 | 802.18 | 3380.57 | strong tie |
| Mainland China | Taiwan | 1170 | 802.18 | 3380.57 | strong tie |
| Mainland China | France | 993 | 802.18 | 3380.57 | strong tie |
| UK | USA | 6710 | 421.63 | 1793.03 | strong tie,super tie |
| UK | Mainland China | 3409 | 421.63 | 1793.03 | strong tie,super tie |
| UK | Germany | 2304 | 421.63 | 1793.03 | strong tie,super tie |
| UK | Australia | 1625 | 421.63 | 1793.03 | strong tie |
| UK | Canada | 1410 | 421.63 | 1793.03 | strong tie |
| UK | France | 1309 | 421.63 | 1793.03 | strong tie |
| UK | Italy | 1002 | 421.63 | 1793.03 | strong tie |
| UK | Netherland | 851 | 421.63 | 1793.03 | strong tie |
| UK | Ireland | 765 | 421.63 | 1793.03 | strong tie |
| UK | Japan | 753 | 421.63 | 1793.03 | strong tie |
| UK | Spain | 703 | 421.63 | 1793.03 | strong tie |
| UK | Scotland | 608 | 421.63 | 1793.03 | strong tie |
| UK | Switzerland | 553 | 421.63 | 1793.03 | strong tie |
| UK | Singapore | 543 | 421.63 | 1793.03 | strong tie |
| UK | India | 535 | 421.63 | 1793.03 | strong tie |
| UK | Greece | 525 | 421.63 | 1793.03 | strong tie |
| UK | Austria | 496 | 421.63 | 1793.03 | strong tie |
| UK | Hong Kong | 428 | 421.63 | 1793.03 | strong tie |
| Germany | USA | 5253 | 364.72 | 1550.41 | strong tie,super tie |
| Germany | UK | 2304 | 364.72 | 1550.41 | strong tie,super tie |
| Germany | Austria | 1602 | 364.72 | 1550.41 | strong tie,super tie |
| Germany | Switzerland | 1555 | 364.72 | 1550.41 | strong tie,super tie |
| Germany | France | 1423 | 364.72 | 1550.41 | strong tie |
| Germany | Mainland China | 1340 | 364.72 | 1550.41 | strong tie |
| Germany | Italy | 1144 | 364.72 | 1550.41 | strong tie |
| Germany | Canada | 1080 | 364.72 | 1550.41 | strong tie |
| Germany | Netherland | 975 | 364.72 | 1550.41 | strong tie |
| Germany | Australia | 943 | 364.72 | 1550.41 | strong tie |
| Germany | Japan | 621 | 364.72 | 1550.41 | strong tie |
| Germany | Denmark | 597 | 364.72 | 1550.41 | strong tie |
| Germany | Spain | 589 | 364.72 | 1550.41 | strong tie |
| Germany | Sweden | 562 | 364.72 | 1550.41 | strong tie |
| Germany | India | 537 | 364.72 | 1550.41 | strong tie |
| Germany | Belgium | 526 | 364.72 | 1550.41 | strong tie |
| Germany | Israel | 390 | 364.72 | 1550.41 | strong tie |
| Canada | USA | 7848 | 373.35 | 1587.22 | strong tie,super tie |
| Canada | Mainland China | 4922 | 373.35 | 1587.22 | strong tie,super tie |
| Canada | UK | 1410 | 373.35 | 1587.22 | strong tie |
| Canada | Germany | 1080 | 373.35 | 1587.22 | strong tie |
| Canada | France | 974 | 373.35 | 1587.22 | strong tie |
| Canada | Australia | 950 | 373.35 | 1587.22 | strong tie |
| Canada | Japan | 779 | 373.35 | 1587.22 | strong tie |
| Canada | India | 777 | 373.35 | 1587.22 | strong tie |
| Canada | Hong Kong | 672 | 373.35 | 1587.22 | strong tie |
| Canada | Singapore | 610 | 373.35 | 1587.22 | strong tie |
| Canada | South Korea | 571 | 373.35 | 1587.22 | strong tie |
| Canada | Taiwan | 484 | 373.35 | 1587.22 | strong tie |
| Canada | Italy | 460 | 373.35 | 1587.22 | strong tie |
| Canada | Iran | 413 | 373.35 | 1587.22 | strong tie |
| Australia | Mainland China | 4554 | 287.78 | 1214.27 | strong tie,super tie |
| Australia | USA | 4076 | 287.78 | 1214.27 | strong tie,super tie |
| Australia | UK | 1625 | 287.78 | 1214.27 | strong tie,super tie |
| Australia | Canada | 950 | 287.78 | 1214.27 | strong tie |
| Australia | Germany | 943 | 287.78 | 1214.27 | strong tie |
| Australia | Singapore | 905 | 287.78 | 1214.27 | strong tie |
| Australia | Hong Kong | 790 | 287.78 | 1214.27 | strong tie |
| Australia | Japan | 510 | 287.78 | 1214.27 | strong tie |
| Australia | Taiwan | 497 | 287.78 | 1214.27 | strong tie |
| Australia | India | 442 | 287.78 | 1214.27 | strong tie |
| Australia | France | 442 | 287.78 | 1214.27 | strong tie |
| Australia | South Korea | 327 | 287.78 | 1214.27 | strong tie |
| Australia | New Zealand | 314 | 287.78 | 1214.27 | strong tie |
| Australia | Netherland | 289 | 287.78 | 1214.27 | strong tie |
| France | USA | 2749 | 248.89 | 1053.14 | strong tie,super tie |
| France | Germany | 1423 | 248.89 | 1053.14 | strong tie,super tie |
| France | UK | 1309 | 248.89 | 1053.14 | strong tie,super tie |
| France | Italy | 1139 | 248.89 | 1053.14 | strong tie,super tie |
| France | Mainland China | 993 | 248.89 | 1053.14 | strong tie |
| France | Canada | 974 | 248.89 | 1053.14 | strong tie |
| France | Spain | 653 | 248.89 | 1053.14 | strong tie |
| France | Switzerland | 614 | 248.89 | 1053.14 | strong tie |
| France | Japan | 552 | 248.89 | 1053.14 | strong tie |
| France | Belgium | 545 | 248.89 | 1053.14 | strong tie |
| France | Australia | 442 | 248.89 | 1053.14 | strong tie |
| France | Netherland | 406 | 248.89 | 1053.14 | strong tie |
| France | Tunisia | 364 | 248.89 | 1053.14 | strong tie |
| France | India | 353 | 248.89 | 1053.14 | strong tie |
| France | Austria | 350 | 248.89 | 1053.14 | strong tie |
| France | South Korea | 329 | 248.89 | 1053.14 | strong tie |
| France | Brazil | 256 | 248.89 | 1053.14 | strong tie |

**S2 Table. Collaboration by low-productivity countries/regions.**

| **Country___*i* /Region___*i*** | **Collaborator** | **Publications** | **<*K_i_*>** | ***Kc*** | **Tie Type** |
| --- | --- | --- | --- | --- | --- |
| Japan | USA | 3435 | 204.77 | 868.63 | strong tie,super tie |
| Japan | Mainland China | 3105 | 204.77 | 868.63 | strong tie,super tie |
| Japan | South Korea | 863 | 204.77 | 868.63 | strong tie |
| Japan | Canada | 779 | 204.77 | 868.63 | strong tie |
| Japan | UK | 753 | 204.77 | 868.63 | strong tie |
| Japan | Germany | 621 | 204.77 | 868.63 | strong tie |
| Japan | France | 552 | 204.77 | 868.63 | strong tie |
| Japan | Australia | 510 | 204.77 | 868.63 | strong tie |
| Japan | India | 411 | 204.77 | 868.63 | strong tie |
| Japan | Singapore | 341 | 204.77 | 868.63 | strong tie |
| Japan | Hong Kong | 317 | 204.77 | 868.63 | strong tie |
| Japan | Taiwan | 252 | 204.77 | 868.63 | strong tie |
| Japan | Italy | 215 | 204.77 | 868.63 | strong tie |
| South Korea | USA | 5375 | 177.47 | 739.35 | strong tie,super tie |
| South Korea | Mainland China | 1254 | 177.47 | 739.35 | strong tie,super tie |
| South Korea | Japan | 863 | 177.47 | 739.35 | strong tie,super tie |
| South Korea | Canada | 571 | 177.47 | 739.35 | strong tie |
| South Korea | UK | 352 | 177.47 | 739.35 | strong tie |
| South Korea | France | 329 | 177.47 | 739.35 | strong tie |
| South Korea | Australia | 327 | 177.47 | 739.35 | strong tie |
| South Korea | Germany | 304 | 177.47 | 739.35 | strong tie |
| South Korea | Singapore | 274 | 177.47 | 739.35 | strong tie |
| South Korea | Hong Kong | 247 | 177.47 | 739.35 | strong tie |
| South Korea | India | 234 | 177.47 | 739.35 | strong tie |
| South Korea | Taiwan | 194 | 177.47 | 739.35 | strong tie |
| India | USA | 5707 | 173.59 | 733.23 | strong tie,super tie |
| India | Canada | 777 | 173.59 | 733.23 | strong tie,super tie |
| India | Mainland China | 548 | 173.59 | 733.23 | strong tie |
| India | Germany | 537 | 173.59 | 733.23 | strong tie |
| India | UK | 535 | 173.59 | 733.23 | strong tie |
| India | Singapore | 461 | 173.59 | 733.23 | strong tie |
| India | Australia | 442 | 173.59 | 733.23 | strong tie |
| India | Japan | 411 | 173.59 | 733.23 | strong tie |
| India | France | 353 | 173.59 | 733.23 | strong tie |
| India | South Korea | 234 | 173.59 | 733.23 | strong tie |
| Singapore | Mainland China | 3551 | 194.35 | 798.00 | strong tie,super tie |
| Singapore | USA | 2695 | 194.35 | 798.00 | strong tie,super tie |
| Singapore | Australia | 905 | 194.35 | 798.00 | strong tie,super tie |
| Singapore | Canada | 610 | 194.35 | 798.00 | strong tie |
| Singapore | Hong Kong | 575 | 194.35 | 798.00 | strong tie |
| Singapore | UK | 543 | 194.35 | 798.00 | strong tie |
| Singapore | India | 461 | 194.35 | 798.00 | strong tie |
| Singapore | Taiwan | 354 | 194.35 | 798.00 | strong tie |
| Singapore | Japan | 341 | 194.35 | 798.00 | strong tie |
| Singapore | South Korea | 274 | 194.35 | 798.00 | strong tie |
| Singapore | France | 242 | 194.35 | 798.00 | strong tie |
| Singapore | Germany | 219 | 194.35 | 798.00 | strong tie |
| Italy | USA | 2407 | 167.16 | 701.12 | strong tie,super tie |
| Italy | Germany | 1144 | 167.16 | 701.12 | strong tie,super tie |
| Italy | France | 1139 | 167.16 | 701.12 | strong tie,super tie |
| Italy | UK | 1002 | 167.16 | 701.12 | strong tie,super tie |
| Italy | Spain | 622 | 167.16 | 701.12 | strong tie |
| Italy | Switzerland | 525 | 167.16 | 701.12 | strong tie |
| Italy | Canada | 460 | 167.16 | 701.12 | strong tie |
| Italy | Netherland | 372 | 167.16 | 701.12 | strong tie |
| Italy | Mainland China | 298 | 167.16 | 701.12 | strong tie |
| Italy | Austria | 297 | 167.16 | 701.12 | strong tie |
| Italy | Belgium | 222 | 167.16 | 701.12 | strong tie |
| Italy | Sweden | 220 | 167.16 | 701.12 | strong tie |
| Italy | Australia | 219 | 167.16 | 701.12 | strong tie |
| Italy | Japan | 215 | 167.16 | 701.12 | strong tie |
| Hong Kong | USA | 3637 | 185.91 | 747.61 | strong tie,super tie |
| Hong Kong | Mainland China | 2591 | 185.91 | 747.61 | strong tie,super tie |
| Hong Kong | Australia | 790 | 185.91 | 747.61 | strong tie,super tie |
| Hong Kong | Canada | 672 | 185.91 | 747.61 | strong tie |
| Hong Kong | Singapore | 575 | 185.91 | 747.61 | strong tie |
| Hong Kong | UK | 428 | 185.91 | 747.61 | strong tie |
| Hong Kong | Japan | 317 | 185.91 | 747.61 | strong tie |
| Hong Kong | South Korea | 247 | 185.91 | 747.61 | strong tie |
| Hong Kong | Germany | 191 | 185.91 | 747.61 | strong tie |
| Switzerland | USA | 1611 | 130.44 | 538.32 | strong tie,super tie |
| Switzerland | Germany | 1555 | 130.44 | 538.32 | strong tie,super tie |
| Switzerland | France | 614 | 130.44 | 538.32 | strong tie,super tie |
| Switzerland | UK | 553 | 130.44 | 538.32 | strong tie,super tie |
| Switzerland | Italy | 525 | 130.44 | 538.32 | strong tie |
| Switzerland | Mainland China | 324 | 130.44 | 538.32 | strong tie |
| Switzerland | Canada | 302 | 130.44 | 538.32 | strong tie |
| Switzerland | Austria | 258 | 130.44 | 538.32 | strong tie |
| Switzerland | Netherland | 222 | 130.44 | 538.32 | strong tie |
| Switzerland | Australia | 222 | 130.44 | 538.32 | strong tie |
| Switzerland | Spain | 196 | 130.44 | 538.32 | strong tie |
| Switzerland | Singapore | 153 | 130.44 | 538.32 | strong tie |
| Switzerland | Belgium | 152 | 130.44 | 538.32 | strong tie |
| Switzerland | Japan | 134 | 130.44 | 538.32 | strong tie |
| Switzerland | India | 132 | 130.44 | 538.32 | strong tie |
| Netherland | USA | 1231 | 123.15 | 511.77 | strong tie,super tie |
| Netherland | Germany | 975 | 123.15 | 511.77 | strong tie,super tie |
| Netherland | UK | 851 | 123.15 | 511.77 | strong tie,super tie |
| Netherland | Belgium | 467 | 123.15 | 511.77 | strong tie |
| Netherland | Mainland China | 434 | 123.15 | 511.77 | strong tie |
| Netherland | France | 406 | 123.15 | 511.77 | strong tie |
| Netherland | Italy | 372 | 123.15 | 511.77 | strong tie |
| Netherland | Canada | 305 | 123.15 | 511.77 | strong tie |
| Netherland | Australia | 289 | 123.15 | 511.77 | strong tie |
| Netherland | Switzerland | 222 | 123.15 | 511.77 | strong tie |
| Netherland | Spain | 201 | 123.15 | 511.77 | strong tie |
| Netherland | Austria | 160 | 123.15 | 511.77 | strong tie |
| Netherland | Sweden | 157 | 123.15 | 511.77 | strong tie |
| Netherland | Japan | 137 | 123.15 | 511.77 | strong tie |
| Netherland | Portugal | 130 | 123.15 | 511.77 | strong tie |
| Netherland | Denmark | 127 | 123.15 | 511.77 | strong tie |
| Netherland | Israel | 126 | 123.15 | 511.77 | strong tie |
| Spain | USA | 1179 | 110.65 | 464.28 | strong tie,super tie |
| Spain | UK | 703 | 110.65 | 464.28 | strong tie,super tie |
| Spain | France | 653 | 110.65 | 464.28 | strong tie,super tie |
| Spain | Italy | 622 | 110.65 | 464.28 | strong tie,super tie |
| Spain | Germany | 589 | 110.65 | 464.28 | strong tie,super tie |
| Spain | Mexico | 292 | 110.65 | 464.28 | strong tie |
| Spain | Mainland China | 252 | 110.65 | 464.28 | strong tie |
| Spain | Canada | 221 | 110.65 | 464.28 | strong tie |
| Spain | Netherland | 201 | 110.65 | 464.28 | strong tie |
| Spain | Japan | 200 | 110.65 | 464.28 | strong tie |
| Spain | Switzerland | 196 | 110.65 | 464.28 | strong tie |
| Spain | Australia | 187 | 110.65 | 464.28 | strong tie |
| Spain | Portugal | 186 | 110.65 | 464.28 | strong tie |
| Spain | Chile | 165 | 110.65 | 464.28 | strong tie |
| Spain | Belgium | 145 | 110.65 | 464.28 | strong tie |
| Spain | Greece | 141 | 110.65 | 464.28 | strong tie |
| Spain | Sweden | 129 | 110.65 | 464.28 | strong tie |
| Spain | Ireland | 123 | 110.65 | 464.28 | strong tie |
| Spain | Austria | 113 | 110.65 | 464.28 | strong tie |
| Taiwan | USA | 2962 | 133.64 | 531.52 | strong tie,super tie |
| Taiwan | Mainland China | 1170 | 133.64 | 531.52 | strong tie,super tie |
| Taiwan | Australia | 497 | 133.64 | 531.52 | strong tie |
| Taiwan | Canada | 484 | 133.64 | 531.52 | strong tie |
| Taiwan | Singapore | 354 | 133.64 | 531.52 | strong tie |
| Taiwan | UK | 347 | 133.64 | 531.52 | strong tie |
| Taiwan | Japan | 252 | 133.64 | 531.52 | strong tie |
| Taiwan | South Korea | 194 | 133.64 | 531.52 | strong tie |
| Taiwan | Germany | 157 | 133.64 | 531.52 | strong tie |
| Austria | Germany | 1602 | 97.26 | 401.83 | strong tie,super tie |
| Austria | USA | 791 | 97.26 | 401.83 | strong tie,super tie |
| Austria | UK | 496 | 97.26 | 401.83 | strong tie,super tie |
| Austria | France | 350 | 97.26 | 401.83 | strong tie |
| Austria | Italy | 297 | 97.26 | 401.83 | strong tie |
| Austria | Switzerland | 258 | 97.26 | 401.83 | strong tie |
| Austria | Australia | 193 | 97.26 | 401.83 | strong tie |
| Austria | Mainland China | 185 | 97.26 | 401.83 | strong tie |
| Austria | Netherland | 160 | 97.26 | 401.83 | strong tie |
| Austria | Japan | 152 | 97.26 | 401.83 | strong tie |
| Austria | Canada | 148 | 97.26 | 401.83 | strong tie |
| Austria | Denmark | 115 | 97.26 | 401.83 | strong tie |
| Austria | Spain | 113 | 97.26 | 401.83 | strong tie |
| Austria | Belgium | 111 | 97.26 | 401.83 | strong tie |
| Austria | Ireland | 104 | 97.26 | 401.83 | strong tie |
| Israel | USA | 2941 | 102.63 | 401.58 | strong tie,super tie |
| Israel | Germany | 390 | 102.63 | 401.58 | strong tie |
| Israel | Canada | 271 | 102.63 | 401.58 | strong tie |
| Israel | UK | 265 | 102.63 | 401.58 | strong tie |
| Israel | France | 161 | 102.63 | 401.58 | strong tie |
| Israel | Italy | 139 | 102.63 | 401.58 | strong tie |
| Israel | Netherland | 126 | 102.63 | 401.58 | strong tie |
| Israel | Switzerland | 117 | 102.63 | 401.58 | strong tie |
| Belgium | USA | 715 | 80.98 | 335.11 | strong tie,super tie |
| Belgium | France | 545 | 80.98 | 335.11 | strong tie,super tie |
| Belgium | Germany | 526 | 80.98 | 335.11 | strong tie,super tie |
| Belgium | Netherland | 467 | 80.98 | 335.11 | strong tie,super tie |
| Belgium | UK | 401 | 80.98 | 335.11 | strong tie,super tie |
| Belgium | Canada | 321 | 80.98 | 335.11 | strong tie |
| Belgium | Mainland China | 282 | 80.98 | 335.11 | strong tie |
| Belgium | Italy | 222 | 80.98 | 335.11 | strong tie |
| Belgium | Switzerland | 152 | 80.98 | 335.11 | strong tie |
| Belgium | Spain | 145 | 80.98 | 335.11 | strong tie |
| Belgium | Japan | 139 | 80.98 | 335.11 | strong tie |
| Belgium | Australia | 134 | 80.98 | 335.11 | strong tie |
| Belgium | Austria | 111 | 80.98 | 335.11 | strong tie |
| Belgium | India | 92 | 80.98 | 335.11 | strong tie |
| Belgium | South Korea | 82 | 80.98 | 335.11 | strong tie |
| Sweden | USA | 858 | 75.70 | 310.68 | strong tie,super tie |
| Sweden | Germany | 562 | 75.70 | 310.68 | strong tie,super tie |
| Sweden | Mainland China | 403 | 75.70 | 310.68 | strong tie,super tie |
| Sweden | UK | 388 | 75.70 | 310.68 | strong tie,super tie |
| Sweden | France | 234 | 75.70 | 310.68 | strong tie |
| Sweden | Italy | 220 | 75.70 | 310.68 | strong tie |
| Sweden | Denmark | 176 | 75.70 | 310.68 | strong tie |
| Sweden | Canada | 166 | 75.70 | 310.68 | strong tie |
| Sweden | Netherland | 157 | 75.70 | 310.68 | strong tie |
| Sweden | Finland | 143 | 75.70 | 310.68 | strong tie |
| Sweden | Spain | 129 | 75.70 | 310.68 | strong tie |
| Sweden | Australia | 129 | 75.70 | 310.68 | strong tie |
| Sweden | Norway | 104 | 75.70 | 310.68 | strong tie |
| Sweden | India | 101 | 75.70 | 310.68 | strong tie |
| Sweden | Switzerland | 100 | 75.70 | 310.68 | strong tie |
| Sweden | Japan | 85 | 75.70 | 310.68 | strong tie |
| Sweden | Austria | 82 | 75.70 | 310.68 | strong tie |
| Ireland | UK | 765 | 60.09 | 246.67 | strong tie,super tie |
| Ireland | USA | 681 | 60.09 | 246.67 | strong tie,super tie |
| Ireland | Germany | 293 | 60.09 | 246.67 | strong tie,super tie |
| Ireland | Mainland China | 254 | 60.09 | 246.67 | strong tie,super tie |
| Ireland | Canada | 210 | 60.09 | 246.67 | strong tie |
| Ireland | France | 193 | 60.09 | 246.67 | strong tie |
| Ireland | Italy | 166 | 60.09 | 246.67 | strong tie |
| Ireland | Australia | 165 | 60.09 | 246.67 | strong tie |
| Ireland | Spain | 123 | 60.09 | 246.67 | strong tie |
| Ireland | Austria | 104 | 60.09 | 246.67 | strong tie |
| Ireland | India | 83 | 60.09 | 246.67 | strong tie |
| Ireland | Netherland | 70 | 60.09 | 246.67 | strong tie |
| Denmark | Germany | 597 | 62.65 | 249.25 | strong tie,super tie |
| Denmark | USA | 572 | 62.65 | 249.25 | strong tie,super tie |
| Denmark | UK | 278 | 62.65 | 249.25 | strong tie,super tie |
| Denmark | Sweden | 176 | 62.65 | 249.25 | strong tie |
| Denmark | Mainland China | 174 | 62.65 | 249.25 | strong tie |
| Denmark | Australia | 164 | 62.65 | 249.25 | strong tie |
| Denmark | France | 136 | 62.65 | 249.25 | strong tie |
| Denmark | Canada | 134 | 62.65 | 249.25 | strong tie |
| Denmark | Netherland | 127 | 62.65 | 249.25 | strong tie |
| Denmark | Italy | 116 | 62.65 | 249.25 | strong tie |
| Denmark | Austria | 115 | 62.65 | 249.25 | strong tie |
| Denmark | Switzerland | 114 | 62.65 | 249.25 | strong tie |
| Denmark | Belgium | 78 | 62.65 | 249.25 | strong tie |
| Denmark | Singapore | 73 | 62.65 | 249.25 | strong tie |
| Denmark | Norway | 69 | 62.65 | 249.25 | strong tie |
| Denmark | Finland | 67 | 62.65 | 249.25 | strong tie |
| Denmark | New Zealand | 67 | 62.65 | 249.25 | strong tie |
| Greece | USA | 920 | 52.83 | 216.36 | strong tie,super tie |
| Greece | UK | 525 | 52.83 | 216.36 | strong tie,super tie |
| Greece | Germany | 252 | 52.83 | 216.36 | strong tie,super tie |
| Greece | France | 156 | 52.83 | 216.36 | strong tie |
| Greece | Spain | 141 | 52.83 | 216.36 | strong tie |
| Greece | Italy | 127 | 52.83 | 216.36 | strong tie |
| Greece | Switzerland | 108 | 52.83 | 216.36 | strong tie |
| Greece | Canada | 105 | 52.83 | 216.36 | strong tie |
| Greece | Mainland China | 103 | 52.83 | 216.36 | strong tie |
| Greece | Cyprus | 76 | 52.83 | 216.36 | strong tie |
| Greece | Netherland | 70 | 52.83 | 216.36 | strong tie |
| Greece | India | 68 | 52.83 | 216.36 | strong tie |
| Greece | Sweden | 63 | 52.83 | 216.36 | strong tie |
| Greece | Romania | 55 | 52.83 | 216.36 | strong tie |
| Finland | USA | 436 | 53.63 | 212.79 | strong tie,super tie |
| Finland | Germany | 293 | 53.63 | 212.79 | strong tie,super tie |
| Finland | UK | 258 | 53.63 | 212.79 | strong tie,super tie |
| Finland | Mainland China | 247 | 53.63 | 212.79 | strong tie,super tie |
| Finland | Sweden | 143 | 53.63 | 212.79 | strong tie |
| Finland | Japan | 117 | 53.63 | 212.79 | strong tie |
| Finland | Canada | 116 | 53.63 | 212.79 | strong tie |
| Finland | Netherland | 115 | 53.63 | 212.79 | strong tie |
| Finland | Spain | 108 | 53.63 | 212.79 | strong tie |
| Finland | Italy | 108 | 53.63 | 212.79 | strong tie |
| Finland | France | 105 | 53.63 | 212.79 | strong tie |
| Finland | Switzerland | 96 | 53.63 | 212.79 | strong tie |
| Finland | Australia | 84 | 53.63 | 212.79 | strong tie |
| Finland | Denmark | 67 | 53.63 | 212.79 | strong tie |
| Finland | Belgium | 57 | 53.63 | 212.79 | strong tie |
| Finland | Poland | 56 | 53.63 | 212.79 | strong tie |
| Brazil | USA | 524 | 48.59 | 194.06 | strong tie,super tie |
| Brazil | France | 256 | 48.59 | 194.06 | strong tie,super tie |
| Brazil | Portugal | 254 | 48.59 | 194.06 | strong tie,super tie |
| Brazil | UK | 235 | 48.59 | 194.06 | strong tie,super tie |
| Brazil | Germany | 208 | 48.59 | 194.06 | strong tie,super tie |
| Brazil | Canada | 190 | 48.59 | 194.06 | strong tie |
| Brazil | Italy | 131 | 48.59 | 194.06 | strong tie |
| Brazil | Spain | 101 | 48.59 | 194.06 | strong tie |
| Brazil | India | 93 | 48.59 | 194.06 | strong tie |
| Brazil | Mainland China | 82 | 48.59 | 194.06 | strong tie |
| Brazil | Japan | 79 | 48.59 | 194.06 | strong tie |
| Brazil | Australia | 69 | 48.59 | 194.06 | strong tie |
| New Zealand | USA | 547 | 48.91 | 192.86 | strong tie,super tie |
| New Zealand | UK | 318 | 48.91 | 192.86 | strong tie,super tie |
| New Zealand | Australia | 314 | 48.91 | 192.86 | strong tie,super tie |
| New Zealand | Mainland China | 225 | 48.91 | 192.86 | strong tie,super tie |
| New Zealand | Canada | 191 | 48.91 | 192.86 | strong tie |
| New Zealand | Germany | 190 | 48.91 | 192.86 | strong tie |
| New Zealand | Singapore | 111 | 48.91 | 192.86 | strong tie |
| New Zealand | Japan | 95 | 48.91 | 192.86 | strong tie |
| New Zealand | South Korea | 74 | 48.91 | 192.86 | strong tie |
| New Zealand | Denmark | 67 | 48.91 | 192.86 | strong tie |
| New Zealand | Netherland | 57 | 48.91 | 192.86 | strong tie |
| New Zealand | Austria | 52 | 48.91 | 192.86 | strong tie |
| Norway | USA | 339 | 44.05 | 176.26 | strong tie,super tie |
| Norway | Germany | 272 | 44.05 | 176.26 | strong tie,super tie |
| Norway | France | 224 | 44.05 | 176.26 | strong tie,super tie |
| Norway | Mainland China | 165 | 44.05 | 176.26 | strong tie |
| Norway | UK | 163 | 44.05 | 176.26 | strong tie |
| Norway | Canada | 156 | 44.05 | 176.26 | strong tie |
| Norway | Australia | 128 | 44.05 | 176.26 | strong tie |
| Norway | Netherland | 108 | 44.05 | 176.26 | strong tie |
| Norway | Sweden | 104 | 44.05 | 176.26 | strong tie |
| Norway | India | 77 | 44.05 | 176.26 | strong tie |
| Norway | Italy | 76 | 44.05 | 176.26 | strong tie |
| Norway | Austria | 72 | 44.05 | 176.26 | strong tie |
| Norway | Denmark | 69 | 44.05 | 176.26 | strong tie |
| Norway | Spain | 56 | 44.05 | 176.26 | strong tie |
| Norway | Luxembourg | 54 | 44.05 | 176.26 | strong tie |
| Portugal | USA | 339 | 36.56 | 146.78 | strong tie,super tie |
| Portugal | Brazil | 254 | 36.56 | 146.78 | strong tie,super tie |
| Portugal | UK | 236 | 36.56 | 146.78 | strong tie,super tie |
| Portugal | Spain | 186 | 36.56 | 146.78 | strong tie,super tie |
| Portugal | Germany | 167 | 36.56 | 146.78 | strong tie,super tie |
| Portugal | Netherland | 130 | 36.56 | 146.78 | strong tie |
| Portugal | France | 129 | 36.56 | 146.78 | strong tie |
| Portugal | Italy | 84 | 36.56 | 146.78 | strong tie |
| Portugal | Canada | 81 | 36.56 | 146.78 | strong tie |
| Portugal | Belgium | 47 | 36.56 | 146.78 | strong tie |
| Portugal | Mainland China | 43 | 36.56 | 146.78 | strong tie |
| Portugal | Japan | 40 | 36.56 | 146.78 | strong tie |
| Turkey | USA | 776 | 31.98 | 128.37 | strong tie,super tie |
| Turkey | UK | 188 | 31.98 | 128.37 | strong tie,super tie |
| Turkey | Netherland | 99 | 31.98 | 128.37 | strong tie |
| Turkey | Canada | 94 | 31.98 | 128.37 | strong tie |
| Turkey | Germany | 88 | 31.98 | 128.37 | strong tie |
| Turkey | India | 72 | 31.98 | 128.37 | strong tie |
| Turkey | Switzerland | 59 | 31.98 | 128.37 | strong tie |
| Turkey | Mainland China | 59 | 31.98 | 128.37 | strong tie |
| Turkey | Italy | 48 | 31.98 | 128.37 | strong tie |
| Turkey | Lebanon | 48 | 31.98 | 128.37 | strong tie |
| Turkey | France | 42 | 31.98 | 128.37 | strong tie |
| Iran | USA | 444 | 39.67 | 150.51 | strong tie,super tie |
| Iran | Canada | 413 | 39.67 | 150.51 | strong tie,super tie |
| Iran | UK | 150 | 39.67 | 150.51 | strong tie |
| Iran | Australia | 150 | 39.67 | 150.51 | strong tie |
| Iran | Netherland | 77 | 39.67 | 150.51 | strong tie |
| Iran | Germany | 71 | 39.67 | 150.51 | strong tie |
| Iran | Austria | 65 | 39.67 | 150.51 | strong tie |
| Iran | Malaysia | 51 | 39.67 | 150.51 | strong tie |
| Iran | Japan | 45 | 39.67 | 150.51 | strong tie |
| Iran | Mainland China | 41 | 39.67 | 150.51 | strong tie |
| Poland | USA | 329 | 32.93 | 130.75 | strong tie,super tie |
| Poland | Germany | 217 | 32.93 | 130.75 | strong tie,super tie |
| Poland | France | 147 | 32.93 | 130.75 | strong tie,super tie |
| Poland | Canada | 133 | 32.93 | 130.75 | strong tie |
| Poland | UK | 126 | 32.93 | 130.75 | strong tie |
| Poland | Italy | 118 | 32.93 | 130.75 | strong tie |
| Poland | Australia | 68 | 32.93 | 130.75 | strong tie |
| Poland | Netherland | 60 | 32.93 | 130.75 | strong tie |
| Poland | Japan | 57 | 32.93 | 130.75 | strong tie |
| Poland | Finland | 56 | 32.93 | 130.75 | strong tie |
| Poland | Mainland China | 55 | 32.93 | 130.75 | strong tie |
| Poland | Switzerland | 52 | 32.93 | 130.75 | strong tie |
| Poland | Spain | 50 | 32.93 | 130.75 | strong tie |
| Poland | Singapore | 43 | 32.93 | 130.75 | strong tie |
| Poland | Russia | 36 | 32.93 | 130.75 | strong tie |
| Poland | Sweden | 35 | 32.93 | 130.75 | strong tie |
| Poland | Austria | 33 | 32.93 | 130.75 | strong tie |
| Poland | Czech Republic | 33 | 32.93 | 130.75 | strong tie |
| Mexico | USA | 404 | 32.39 | 122.16 | strong tie,super tie |
| Mexico | Spain | 292 | 32.39 | 122.16 | strong tie,super tie |
| Mexico | France | 167 | 32.39 | 122.16 | strong tie,super tie |
| Mexico | UK | 93 | 32.39 | 122.16 | strong tie |
| Mexico | Germany | 59 | 32.39 | 122.16 | strong tie |
| Mexico | Canada | 50 | 32.39 | 122.16 | strong tie |
| Mexico | Mainland China | 43 | 32.39 | 122.16 | strong tie |
| Mexico | Japan | 38 | 32.39 | 122.16 | strong tie |
| Mexico | Brazil | 37 | 32.39 | 122.16 | strong tie |
| Mexico | Australia | 37 | 32.39 | 122.16 | strong tie |
| Mexico | Italy | 34 | 32.39 | 122.16 | strong tie |
| Malaysia | USA | 222 | 29.75 | 114.16 | strong tie,super tie |
| Malaysia | Australia | 185 | 29.75 | 114.16 | strong tie,super tie |
| Malaysia | UK | 177 | 29.75 | 114.16 | strong tie,super tie |
| Malaysia | India | 103 | 29.75 | 114.16 | strong tie |
| Malaysia | Japan | 93 | 29.75 | 114.16 | strong tie |
| Malaysia | Singapore | 92 | 29.75 | 114.16 | strong tie |
| Malaysia | Mainland China | 90 | 29.75 | 114.16 | strong tie |
| Malaysia | South Korea | 69 | 29.75 | 114.16 | strong tie |
| Malaysia | Canada | 57 | 29.75 | 114.16 | strong tie |
| Malaysia | Iran | 51 | 29.75 | 114.16 | strong tie |
| Malaysia | France | 39 | 29.75 | 114.16 | strong tie |
| Malaysia | Hong Kong | 38 | 29.75 | 114.16 | strong tie |
| Malaysia | New Zealand | 35 | 29.75 | 114.16 | strong tie |
| Malaysia | Pakistan | 33 | 29.75 | 114.16 | strong tie |
| Malaysia | Saudi Arabia | 32 | 29.75 | 114.16 | strong tie |
| Scotland | UK | 608 | 35.09 | 130.51 | strong tie,super tie |
| Scotland | USA | 254 | 35.09 | 130.51 | strong tie,super tie |
| Scotland | Mainland China | 144 | 35.09 | 130.51 | strong tie,super tie |
| Scotland | Germany | 106 | 35.09 | 130.51 | strong tie |
| Scotland | Australia | 66 | 35.09 | 130.51 | strong tie |
| Scotland | France | 38 | 35.09 | 130.51 | strong tie |
| Saudi Arabia | USA | 189 | 27.11 | 103.68 | strong tie,super tie |
| Saudi Arabia | Canada | 154 | 27.11 | 103.68 | strong tie,super tie |
| Saudi Arabia | UK | 113 | 27.11 | 103.68 | strong tie,super tie |
| Saudi Arabia | Mainland China | 94 | 27.11 | 103.68 | strong tie |
| Saudi Arabia | Australia | 87 | 27.11 | 103.68 | strong tie |
| Saudi Arabia | France | 79 | 27.11 | 103.68 | strong tie |
| Saudi Arabia | Egypt | 60 | 27.11 | 103.68 | strong tie |
| Saudi Arabia | Japan | 53 | 27.11 | 103.68 | strong tie |
| Saudi Arabia | Germany | 49 | 27.11 | 103.68 | strong tie |
| Saudi Arabia | Pakistan | 46 | 27.11 | 103.68 | strong tie |
| Saudi Arabia | India | 44 | 27.11 | 103.68 | strong tie |
| Saudi Arabia | South Korea | 41 | 27.11 | 103.68 | strong tie |
| Saudi Arabia | Malaysia | 32 | 27.11 | 103.68 | strong tie |
| Saudi Arabia | Netherland | 31 | 27.11 | 103.68 | strong tie |
| Saudi Arabia | Austria | 29 | 27.11 | 103.68 | strong tie |
| Russia | USA | 199 | 18.13 | 70.42 | strong tie,super tie |
| Russia | Germany | 96 | 18.13 | 70.42 | strong tie,super tie |
| Russia | UK | 92 | 18.13 | 70.42 | strong tie,super tie |
| Russia | France | 83 | 18.13 | 70.42 | strong tie,super tie |
| Russia | Netherland | 62 | 18.13 | 70.42 | strong tie |
| Russia | Italy | 54 | 18.13 | 70.42 | strong tie |
| Russia | Japan | 44 | 18.13 | 70.42 | strong tie |
| Russia | Finland | 39 | 18.13 | 70.42 | strong tie |
| Russia | Ukraine | 36 | 18.13 | 70.42 | strong tie |
| Russia | Poland | 36 | 18.13 | 70.42 | strong tie |
| Russia | Canada | 32 | 18.13 | 70.42 | strong tie |
| Russia | Mainland China | 31 | 18.13 | 70.42 | strong tie |
| Russia | South Korea | 28 | 18.13 | 70.42 | strong tie |
| Russia | Belgium | 25 | 18.13 | 70.42 | strong tie |
| Russia | Sweden | 20 | 18.13 | 70.42 | strong tie |
| Romania | USA | 144 | 19.40 | 73.04 | strong tie,super tie |
| Romania | France | 101 | 19.40 | 73.04 | strong tie,super tie |
| Romania | UK | 74 | 19.40 | 73.04 | strong tie,super tie |
| Romania | Germany | 72 | 19.40 | 73.04 | strong tie |
| Romania | Greece | 55 | 19.40 | 73.04 | strong tie |
| Romania | Austria | 53 | 19.40 | 73.04 | strong tie |
| Romania | Italy | 52 | 19.40 | 73.04 | strong tie |
| Romania | Mainland China | 35 | 19.40 | 73.04 | strong tie |
| Romania | Spain | 33 | 19.40 | 73.04 | strong tie |
| Romania | Hungary | 32 | 19.40 | 73.04 | strong tie |
| Romania | Canada | 30 | 19.40 | 73.04 | strong tie |
| Romania | Netherland | 26 | 19.40 | 73.04 | strong tie |
| Romania | Belgium | 25 | 19.40 | 73.04 | strong tie |
| Romania | Norway | 22 | 19.40 | 73.04 | strong tie |
| Romania | Switzerland | 21 | 19.40 | 73.04 | strong tie |
| Romania | India | 21 | 19.40 | 73.04 | strong tie |
| Romania | Poland | 21 | 19.40 | 73.04 | strong tie |
| Romania | Australia | 21 | 19.40 | 73.04 | strong tie |
| Pakistan | USA | 164 | 20.72 | 78.67 | strong tie,super tie |
| Pakistan | Mainland China | 113 | 20.72 | 78.67 | strong tie,super tie |
| Pakistan | UK | 87 | 20.72 | 78.67 | strong tie,super tie |
| Pakistan | France | 67 | 20.72 | 78.67 | strong tie |
| Pakistan | South Korea | 64 | 20.72 | 78.67 | strong tie |
| Pakistan | Canada | 54 | 20.72 | 78.67 | strong tie |
| Pakistan | Germany | 54 | 20.72 | 78.67 | strong tie |
| Pakistan | Australia | 49 | 20.72 | 78.67 | strong tie |
| Pakistan | Saudi Arabia | 46 | 20.72 | 78.67 | strong tie |
| Pakistan | Sweden | 36 | 20.72 | 78.67 | strong tie |
| Pakistan | Japan | 36 | 20.72 | 78.67 | strong tie |
| Pakistan | Austria | 35 | 20.72 | 78.67 | strong tie |
| Pakistan | Malaysia | 33 | 20.72 | 78.67 | strong tie |
| Pakistan | Netherland | 31 | 20.72 | 78.67 | strong tie |
| Pakistan | Italy | 30 | 20.72 | 78.67 | strong tie |
| Pakistan | India | 26 | 20.72 | 78.67 | strong tie |
| Chile | Spain | 165 | 24.37 | 86.77 | strong tie,super tie |
| Chile | France | 139 | 24.37 | 86.77 | strong tie,super tie |
| Chile | USA | 117 | 24.37 | 86.77 | strong tie,super tie |
| Chile | Canada | 82 | 24.37 | 86.77 | strong tie |
| Chile | Germany | 66 | 24.37 | 86.77 | strong tie |
| Chile | UK | 48 | 24.37 | 86.77 | strong tie |
| Chile | Brazil | 44 | 24.37 | 86.77 | strong tie |
| Chile | Argentina | 40 | 24.37 | 86.77 | strong tie |
| Chile | Finland | 32 | 24.37 | 86.77 | strong tie |
| Chile | Switzerland | 27 | 24.37 | 86.77 | strong tie |
| Chile | Italy | 25 | 24.37 | 86.77 | strong tie |
| Czech Republic | USA | 118 | 19.10 | 71.16 | strong tie,super tie |
| Czech Republic | Germany | 116 | 19.10 | 71.16 | strong tie,super tie |
| Czech Republic | UK | 68 | 19.10 | 71.16 | strong tie |
| Czech Republic | Spain | 66 | 19.10 | 71.16 | strong tie |
| Czech Republic | Slovakia | 63 | 19.10 | 71.16 | strong tie |
| Czech Republic | Austria | 62 | 19.10 | 71.16 | strong tie |
| Czech Republic | France | 53 | 19.10 | 71.16 | strong tie |
| Czech Republic | Switzerland | 35 | 19.10 | 71.16 | strong tie |
| Czech Republic | Poland | 33 | 19.10 | 71.16 | strong tie |
| Czech Republic | Belgium | 28 | 19.10 | 71.16 | strong tie |
| Czech Republic | Sweden | 27 | 19.10 | 71.16 | strong tie |
| Czech Republic | Australia | 27 | 19.10 | 71.16 | strong tie |
| Czech Republic | Italy | 23 | 19.10 | 71.16 | strong tie |
| Czech Republic | Ireland | 22 | 19.10 | 71.16 | strong tie |
| Czech Republic | Netherland | 21 | 19.10 | 71.16 | strong tie |
| Egypt | USA | 296 | 18.19 | 68.24 | strong tie,super tie |
| Egypt | Canada | 114 | 18.19 | 68.24 | strong tie,super tie |
| Egypt | Japan | 82 | 18.19 | 68.24 | strong tie,super tie |
| Egypt | Saudi Arabia | 60 | 18.19 | 68.24 | strong tie |
| Egypt | UK | 52 | 18.19 | 68.24 | strong tie |
| Egypt | Germany | 49 | 18.19 | 68.24 | strong tie |
| Egypt | Mainland China | 35 | 18.19 | 68.24 | strong tie |
| Egypt | France | 32 | 18.19 | 68.24 | strong tie |
| Egypt | The United Arab Emirates | 28 | 18.19 | 68.24 | strong tie |
| Egypt | Australia | 21 | 18.19 | 68.24 | strong tie |
| Thailand | USA | 243 | 20.50 | 72.88 | strong tie,super tie |
| Thailand | Japan | 163 | 20.50 | 72.88 | strong tie,super tie |
| Thailand | Australia | 91 | 20.50 | 72.88 | strong tie,super tie |
| Thailand | UK | 58 | 20.50 | 72.88 | strong tie |
| Thailand | Mainland China | 45 | 20.50 | 72.88 | strong tie |
| Thailand | Germany | 32 | 20.50 | 72.88 | strong tie |
| Thailand | India | 26 | 20.50 | 72.88 | strong tie |
| Thailand | Canada | 26 | 20.50 | 72.88 | strong tie |
| Thailand | France | 23 | 20.50 | 72.88 | strong tie |
| Colombia | USA | 327 | 21.10 | 74.15 | strong tie,super tie |
| Colombia | Spain | 94 | 21.10 | 74.15 | strong tie,super tie |
| Colombia | Mainland China | 80 | 21.10 | 74.15 | strong tie,super tie |
| Colombia | Canada | 33 | 21.10 | 74.15 | strong tie |
| Colombia | France | 31 | 21.10 | 74.15 | strong tie |
| Colombia | UK | 30 | 21.10 | 74.15 | strong tie |
| Colombia | Germany | 26 | 21.10 | 74.15 | strong tie |
| Vietnam | France | 135 | 17.00 | 59.80 | strong tie,super tie |
| Vietnam | USA | 105 | 17.00 | 59.80 | strong tie,super tie |
| Vietnam | Japan | 74 | 17.00 | 59.80 | strong tie,super tie |
| Vietnam | South Korea | 58 | 17.00 | 59.80 | strong tie |
| Vietnam | Australia | 35 | 17.00 | 59.80 | strong tie |
| Vietnam | Mainland China | 34 | 17.00 | 59.80 | strong tie |
| Vietnam | Germany | 34 | 17.00 | 59.80 | strong tie |
| Vietnam | UK | 23 | 17.00 | 59.80 | strong tie |
| Vietnam | Taiwan | 19 | 17.00 | 59.80 | strong tie |
| Vietnam | Italy | 18 | 17.00 | 59.80 | strong tie |
| Vietnam | Canada | 17 | 17.00 | 59.80 | strong tie |
| Luxembourg | France | 135 | 23.00 | 78.84 | strong tie,super tie |
| Luxembourg | Germany | 127 | 23.00 | 78.84 | strong tie,super tie |
| Luxembourg | USA | 68 | 23.00 | 78.84 | strong tie |
| Luxembourg | UK | 58 | 23.00 | 78.84 | strong tie |
| Luxembourg | Netherland | 57 | 23.00 | 78.84 | strong tie |
| Luxembourg | Norway | 54 | 23.00 | 78.84 | strong tie |
| Luxembourg | Italy | 53 | 23.00 | 78.84 | strong tie |
| Luxembourg | Canada | 41 | 23.00 | 78.84 | strong tie |
| Luxembourg | Belgium | 30 | 23.00 | 78.84 | strong tie |
| Luxembourg | Australia | 30 | 23.00 | 78.84 | strong tie |
| Luxembourg | Mainland China | 29 | 23.00 | 78.84 | strong tie |
| Luxembourg | Sweden | 28 | 23.00 | 78.84 | strong tie |
| Tunisia | France | 364 | 15.93 | 55.80 | strong tie,super tie |
| Tunisia | Canada | 62 | 15.93 | 55.80 | strong tie |
| Tunisia | USA | 34 | 15.93 | 55.80 | strong tie |
| Tunisia | Saudi Arabia | 25 | 15.93 | 55.80 | strong tie |
| Tunisia | Germany | 16 | 15.93 | 55.80 | strong tie |
| The United Arab Emirates | USA | 180 | 16.33 | 56.53 | strong tie,super tie |
| The United Arab Emirates | Canada | 117 | 16.33 | 56.53 | strong tie,super tie |
| The United Arab Emirates | UK | 71 | 16.33 | 56.53 | strong tie,super tie |
| The United Arab Emirates | Egypt | 28 | 16.33 | 56.53 | strong tie |
| The United Arab Emirates | Germany | 28 | 16.33 | 56.53 | strong tie |
| The United Arab Emirates | Australia | 24 | 16.33 | 56.53 | strong tie |
| The United Arab Emirates | Mainland China | 19 | 16.33 | 56.53 | strong tie |
| Hungary | USA | 100 | 15.49 | 54.49 | strong tie,super tie |
| Hungary | Germany | 82 | 15.49 | 54.49 | strong tie,super tie |
| Hungary | UK | 69 | 15.49 | 54.49 | strong tie,super tie |
| Hungary | France | 59 | 15.49 | 54.49 | strong tie,super tie |
| Hungary | Italy | 42 | 15.49 | 54.49 | strong tie |
| Hungary | Romania | 32 | 15.49 | 54.49 | strong tie |
| Hungary | Canada | 28 | 15.49 | 54.49 | strong tie |
| Hungary | Austria | 26 | 15.49 | 54.49 | strong tie |
| Hungary | Switzerland | 21 | 15.49 | 54.49 | strong tie |
| Hungary | Netherland | 19 | 15.49 | 54.49 | strong tie |
| Hungary | Spain | 18 | 15.49 | 54.49 | strong tie |
| South Africa | USA | 179 | 15.35 | 52.94 | strong tie,super tie |
| South Africa | UK | 63 | 15.35 | 52.94 | strong tie,super tie |
| South Africa | Australia | 55 | 15.35 | 52.94 | strong tie,super tie |
| South Africa | Mainland China | 47 | 15.35 | 52.94 | strong tie |
| South Africa | Germany | 44 | 15.35 | 52.94 | strong tie |
| South Africa | Netherland | 33 | 15.35 | 52.94 | strong tie |
| South Africa | Canada | 32 | 15.35 | 52.94 | strong tie |
| South Africa | France | 21 | 15.35 | 52.94 | strong tie |
| South Africa | Finland | 17 | 15.35 | 52.94 | strong tie |
| South Africa | India | 16 | 15.35 | 52.94 | strong tie |
| Qatar | USA | 201 | 16.50 | 57.18 | strong tie,super tie |
| Qatar | Italy | 47 | 16.50 | 57.18 | strong tie |
| Qatar | France | 44 | 16.50 | 57.18 | strong tie |
| Qatar | Germany | 43 | 16.50 | 57.18 | strong tie |
| Qatar | Australia | 32 | 16.50 | 57.18 | strong tie |
| Qatar | Spain | 29 | 16.50 | 57.18 | strong tie |
| Qatar | Mainland China | 25 | 16.50 | 57.18 | strong tie |
| Qatar | Canada | 25 | 16.50 | 57.18 | strong tie |
| Argentina | Spain | 87 | 13.84 | 46.71 | strong tie,super tie |
| Argentina | USA | 62 | 13.84 | 46.71 | strong tie,super tie |
| Argentina | France | 61 | 13.84 | 46.71 | strong tie,super tie |
| Argentina | Germany | 51 | 13.84 | 46.71 | strong tie,super tie |
| Argentina | Chile | 40 | 13.84 | 46.71 | strong tie |
| Argentina | UK | 35 | 13.84 | 46.71 | strong tie |
| Argentina | Italy | 31 | 13.84 | 46.71 | strong tie |
| Argentina | Canada | 24 | 13.84 | 46.71 | strong tie |
| Argentina | Austria | 17 | 13.84 | 46.71 | strong tie |
| Argentina | Brazil | 17 | 13.84 | 46.71 | strong tie |
| Argentina | Belgium | 15 | 13.84 | 46.71 | strong tie |
| Cyprus | USA | 106 | 12.74 | 42.69 | strong tie,super tie |
| Cyprus | UK | 103 | 12.74 | 42.69 | strong tie,super tie |
| Cyprus | Greece | 76 | 12.74 | 42.69 | strong tie,super tie |
| Cyprus | Germany | 32 | 12.74 | 42.69 | strong tie |
| Cyprus | Turkey | 24 | 12.74 | 42.69 | strong tie |
| Cyprus | Spain | 20 | 12.74 | 42.69 | strong tie |
| Cyprus | Australia | 16 | 12.74 | 42.69 | strong tie |
| Cyprus | Mainland China | 13 | 12.74 | 42.69 | strong tie |
| Lebanon | USA | 89 | 12.30 | 40.79 | strong tie,super tie |
| Lebanon | Canada | 87 | 12.30 | 40.79 | strong tie,super tie |
| Lebanon | France | 71 | 12.30 | 40.79 | strong tie,super tie |
| Lebanon | Turkey | 48 | 12.30 | 40.79 | strong tie |
| Lebanon | Germany | 28 | 12.30 | 40.79 | strong tie |
| Lebanon | Denmark | 20 | 12.30 | 40.79 | strong tie |
| Bangladesh | South Korea | 77 | 12.53 | 40.66 | strong tie,super tie |
| Bangladesh | Japan | 55 | 12.53 | 40.66 | strong tie,super tie |
| Bangladesh | Canada | 53 | 12.53 | 40.66 | strong tie,super tie |
| Bangladesh | Australia | 52 | 12.53 | 40.66 | strong tie,super tie |
| Bangladesh | USA | 51 | 12.53 | 40.66 | strong tie,super tie |
| Bangladesh | India | 30 | 12.53 | 40.66 | strong tie |
| Bangladesh | UK | 29 | 12.53 | 40.66 | strong tie |
| Jordan | USA | 80 | 8.87 | 28.84 | strong tie,super tie |
| Jordan | Canada | 51 | 8.87 | 28.84 | strong tie,super tie |
| Jordan | UK | 40 | 8.87 | 28.84 | strong tie,super tie |
| Jordan | Saudi Arabia | 23 | 8.87 | 28.84 | strong tie |
| Jordan | Japan | 18 | 8.87 | 28.84 | strong tie |
| Jordan | Malaysia | 16 | 8.87 | 28.84 | strong tie |
| Jordan | France | 10 | 8.87 | 28.84 | strong tie |
| Jordan | Australia | 10 | 8.87 | 28.84 | strong tie |
| Slovenia | USA | 62 | 9.85 | 32.65 | strong tie,super tie |
| Slovenia | UK | 43 | 9.85 | 32.65 | strong tie,super tie |
| Slovenia | Germany | 30 | 9.85 | 32.65 | strong tie |
| Slovenia | Austria | 29 | 9.85 | 32.65 | strong tie |
| Slovenia | France | 25 | 9.85 | 32.65 | strong tie |
| Slovenia | Italy | 23 | 9.85 | 32.65 | strong tie |
| Slovenia | Mainland China | 15 | 9.85 | 32.65 | strong tie |
| Slovenia | Spain | 14 | 9.85 | 32.65 | strong tie |
| Slovenia | Canada | 13 | 9.85 | 32.65 | strong tie |
| Slovenia | Portugal | 10 | 9.85 | 32.65 | strong tie |
| Algeria | France | 185 | 11.16 | 35.20 | strong tie,super tie |
| Algeria | UK | 27 | 11.16 | 35.20 | strong tie |
| Algeria | Canada | 26 | 11.16 | 35.20 | strong tie |
| Algeria | USA | 22 | 11.16 | 35.20 | strong tie |
| Algeria | Saudi Arabia | 13 | 11.16 | 35.20 | strong tie |
| Algeria | The United Arab Emirates | 12 | 11.16 | 35.20 | strong tie |
| Ukraine | Mainland China | 55 | 8.00 | 25.82 | strong tie,super tie |
| Ukraine | USA | 38 | 8.00 | 25.82 | strong tie,super tie |
| Ukraine | Russia | 36 | 8.00 | 25.82 | strong tie,super tie |
| Ukraine | Israel | 20 | 8.00 | 25.82 | strong tie |
| Ukraine | Germany | 17 | 8.00 | 25.82 | strong tie |
| Ukraine | Mexico | 16 | 8.00 | 25.82 | strong tie |
| Ukraine | Poland | 13 | 8.00 | 25.82 | strong tie |
| Ukraine | Italy | 12 | 8.00 | 25.82 | strong tie |
| Ukraine | Canada | 12 | 8.00 | 25.82 | strong tie |
| Ukraine | UK | 11 | 8.00 | 25.82 | strong tie |
| Ukraine | Singapore | 9 | 8.00 | 25.82 | strong tie |
| Ukraine | South Korea | 9 | 8.00 | 25.82 | strong tie |
| Ukraine | France | 8 | 8.00 | 25.82 | strong tie |
| Serbia | USA | 51 | 8.41 | 27.15 | strong tie,super tie |
| Serbia | UK | 44 | 8.41 | 27.15 | strong tie,super tie |
| Serbia | Germany | 26 | 8.41 | 27.15 | strong tie |
| Serbia | Bulgaria | 24 | 8.41 | 27.15 | strong tie |
| Serbia | Canada | 17 | 8.41 | 27.15 | strong tie |
| Serbia | Sweden | 17 | 8.41 | 27.15 | strong tie |
| Serbia | Switzerland | 16 | 8.41 | 27.15 | strong tie |
| Serbia | France | 14 | 8.41 | 27.15 | strong tie |
| Serbia | Mainland China | 12 | 8.41 | 27.15 | strong tie |
| Serbia | Italy | 10 | 8.41 | 27.15 | strong tie |
| Serbia | Austria | 9 | 8.41 | 27.15 | strong tie |
| Slovakia | Czech Republic | 63 | 8.24 | 27.05 | strong tie,super tie |
| Slovakia | Germany | 31 | 8.24 | 27.05 | strong tie,super tie |
| Slovakia | USA | 24 | 8.24 | 27.05 | strong tie |
| Slovakia | Finland | 24 | 8.24 | 27.05 | strong tie |
| Slovakia | Switzerland | 19 | 8.24 | 27.05 | strong tie |
| Slovakia | France | 16 | 8.24 | 27.05 | strong tie |
| Slovakia | Austria | 15 | 8.24 | 27.05 | strong tie |
| Slovakia | Canada | 15 | 8.24 | 27.05 | strong tie |
| Slovakia | Australia | 15 | 8.24 | 27.05 | strong tie |
| Slovakia | UK | 12 | 8.24 | 27.05 | strong tie |
| Slovakia | Colombia | 12 | 8.24 | 27.05 | strong tie |
| Slovakia | Italy | 9 | 8.24 | 27.05 | strong tie |
| Bulgaria | USA | 26 | 7.28 | 23.01 | strong tie,super tie |
| Bulgaria | Greece | 26 | 7.28 | 23.01 | strong tie,super tie |
| Bulgaria | Serbia | 24 | 7.28 | 23.01 | strong tie,super tie |
| Bulgaria | Germany | 23 | 7.28 | 23.01 | strong tie |
| Bulgaria | UK | 19 | 7.28 | 23.01 | strong tie |
| Bulgaria | Austria | 14 | 7.28 | 23.01 | strong tie |
| Bulgaria | Belgium | 13 | 7.28 | 23.01 | strong tie |
| Bulgaria | Sweden | 12 | 7.28 | 23.01 | strong tie |
| Bulgaria | France | 12 | 7.28 | 23.01 | strong tie |
| Bulgaria | Romania | 11 | 7.28 | 23.01 | strong tie |
| Bulgaria | Netherland | 11 | 7.28 | 23.01 | strong tie |
| Estonia | Germany | 61 | 9.57 | 29.14 | strong tie,super tie |
| Estonia | Finland | 28 | 9.57 | 29.14 | strong tie |
| Estonia | Netherland | 23 | 9.57 | 29.14 | strong tie |
| Estonia | Australia | 20 | 9.57 | 29.14 | strong tie |
| Estonia | Canada | 19 | 9.57 | 29.14 | strong tie |
| Estonia | USA | 17 | 9.57 | 29.14 | strong tie |
| Estonia | Mainland China | 15 | 9.57 | 29.14 | strong tie |
| Estonia | Italy | 13 | 9.57 | 29.14 | strong tie |
| Estonia | Switzerland | 12 | 9.57 | 29.14 | strong tie |
| Estonia | Austria | 11 | 9.57 | 29.14 | strong tie |
| Oman | UK | 35 | 8.61 | 25.35 | strong tie,super tie |
| Oman | Iran | 35 | 8.61 | 25.35 | strong tie,super tie |
| Oman | India | 30 | 8.61 | 25.35 | strong tie,super tie |
| Oman | Australia | 24 | 8.61 | 25.35 | strong tie |
| Oman | Canada | 22 | 8.61 | 25.35 | strong tie |
| Oman | USA | 17 | 8.61 | 25.35 | strong tie |
| Oman | Mainland China | 10 | 8.61 | 25.35 | strong tie |
| Oman | Germany | 10 | 8.61 | 25.35 | strong tie |
| Kuwait | USA | 58 | 8.64 | 25.47 | strong tie,super tie |
| Kuwait | Australia | 20 | 8.64 | 25.47 | strong tie |
| Kuwait | India | 19 | 8.64 | 25.47 | strong tie |
| Kuwait | Canada | 19 | 8.64 | 25.47 | strong tie |
| Kuwait | Germany | 19 | 8.64 | 25.47 | strong tie |
| Kuwait | UK | 14 | 8.64 | 25.47 | strong tie |
| Kuwait | Turkey | 11 | 8.64 | 25.47 | strong tie |
| Kuwait | Saudi Arabia | 11 | 8.64 | 25.47 | strong tie |
| Kuwait | Egypt | 10 | 8.64 | 25.47 | strong tie |
| Kuwait | The United Arab Emirates | 10 | 8.64 | 25.47 | strong tie |
| Indonesia | Japan | 34 | 6.35 | 18.39 | strong tie,super tie |
| Indonesia | Australia | 33 | 6.35 | 18.39 | strong tie,super tie |
| Indonesia | Malaysia | 15 | 6.35 | 18.39 | strong tie |
| Indonesia | South Korea | 14 | 6.35 | 18.39 | strong tie |
| Indonesia | Germany | 12 | 6.35 | 18.39 | strong tie |
| Indonesia | USA | 11 | 6.35 | 18.39 | strong tie |
| Indonesia | Belgium | 11 | 6.35 | 18.39 | strong tie |
| Indonesia | Austria | 9 | 6.35 | 18.39 | strong tie |
| Indonesia | Netherland | 9 | 6.35 | 18.39 | strong tie |
| Indonesia | UK | 8 | 6.35 | 18.39 | strong tie |
| Morocco | France | 103 | 8.22 | 22.63 | strong tie,super tie |
| Morocco | USA | 16 | 8.22 | 22.63 | strong tie |
| Morocco | Canada | 11 | 8.22 | 22.63 | strong tie |
| Morocco | Saudi Arabia | 10 | 8.22 | 22.63 | strong tie |
| Cuba | Spain | 93 | 6.67 | 18.68 | strong tie,super tie |
| Cuba | Belgium | 13 | 6.67 | 18.68 | strong tie |
| Cuba | Mexico | 11 | 6.67 | 18.68 | strong tie |
| Cuba | Italy | 9 | 6.67 | 18.68 | strong tie |
| Cuba | Canada | 9 | 6.67 | 18.68 | strong tie |
| Cuba | France | 7 | 6.67 | 18.68 | strong tie |
| Macau | Mainland China | 65 | 11.91 | 34.22 | strong tie,super tie |
| Macau | USA | 58 | 11.91 | 34.22 | strong tie,super tie |
| Macau | Australia | 23 | 11.91 | 34.22 | strong tie |
| Macau | UK | 19 | 11.91 | 34.22 | strong tie |
| Macau | Hong Kong | 19 | 11.91 | 34.22 | strong tie |
| Macau | Brazil | 15 | 11.91 | 34.22 | strong tie |
| Macau | Singapore | 14 | 11.91 | 34.22 | strong tie |
| Venezuela | Spain | 47 | 7.25 | 19.86 | strong tie,super tie |
| Venezuela | USA | 40 | 7.25 | 19.86 | strong tie,super tie |
| Venezuela | France | 12 | 7.25 | 19.86 | strong tie |
| Venezuela | Mexico | 11 | 7.25 | 19.86 | strong tie |
| Venezuela | Chile | 9 | 7.25 | 19.86 | strong tie |
| Croatia | USA | 25 | 5.60 | 15.65 | strong tie,super tie |
| Croatia | Germany | 22 | 5.60 | 15.65 | strong tie,super tie |
| Croatia | UK | 19 | 5.60 | 15.65 | strong tie,super tie |
| Croatia | Austria | 15 | 5.60 | 15.65 | strong tie |
| Croatia | France | 11 | 5.60 | 15.65 | strong tie |
| Croatia | Sweden | 9 | 5.60 | 15.65 | strong tie |
| Croatia | Slovenia | 9 | 5.60 | 15.65 | strong tie |
| Croatia | Italy | 7 | 5.60 | 15.65 | strong tie |
| Croatia | Switzerland | 6 | 5.60 | 15.65 | strong tie |
| Croatia | Netherland | 6 | 5.60 | 15.65 | strong tie |
| Iceland | USA | 28 | 5.23 | 13.78 | strong tie,super tie |
| Iceland | UK | 14 | 5.23 | 13.78 | strong tie,super tie |
| Iceland | Germany | 13 | 5.23 | 13.78 | strong tie |
| Iceland | Canada | 11 | 5.23 | 13.78 | strong tie |
| Iceland | Australia | 10 | 5.23 | 13.78 | strong tie |
| Iceland | Finland | 9 | 5.23 | 13.78 | strong tie |
| Iceland | Netherland | 9 | 5.23 | 13.78 | strong tie |
| Iceland | France | 6 | 5.23 | 13.78 | strong tie |

**S3 Table. Parameter estimates for the productivity model of strong ties in 1993-2003.**

| Dataset | *A* | $\rho_{i,t}$ | $ln\bar{c_{i,t}}$ | $ln\bar{a_{i,t}}$ | $lnN_{i,t}$ | *N_obs._* | *Adj.R^2^* |
| --- | --- | --- | --- | --- | --- | --- | --- |
| All | 72 | 0.033 | -0.781 | 0.657 | 0.528 | 1224 | 0.27 |
| *p-value* |  | **0.027** | **0.012** | **0.000** | **0.000** |  |  |
| High-productivity | 7 | 0.003 | -3.294 | 3.675 | 0.273 | 133 | 0.00 |
| *p-value* |  | 0.985 | 0.360 | **0.021** | **0.000** |  |  |
| Low-productivity | 65 | 0.033 | -0.767 | 0.603 | 0.536 | 1091 | 0.32 |
| *p-value* |  | **0.030** | **0.015** | **0.001** | **0.000** |  |  |

Notes: Values significant at the p≤ 0.05 level are indicated in boldface. “All” indicates the combination of all datasets.

**S4 Table. Parameter estimates for the productivity model of strong ties in 2004-2013.**

| Dataset | *A* | $\rho_{i,t}$ | $ln\bar{c_{i,t}}$ | $ln\bar{a_{i,t}}$ | $lnN_{i,t}$ | *N_obs._* | *Adj.R^2^* |
| --- | --- | --- | --- | --- | --- | --- | --- |
| All | 72 | 0.033 | -0.781 | 0.657 | 0.528 | 1224 | 0.27 |
| *p-value* |  | **0.027** | **0.012** | **0.000** | **0.000** |  |  |
| High-productivity | 7 | 0.003 | -3.294 | 3.675 | 0.273 | 133 | 0.00 |
| *p-value* |  | 0.985 | 0.360 | **0.021** | **0.000** |  |  |
| Low-productivity | 65 | 0.033 | -0.767 | 0.603 | 0.536 | 1091 | 0.32 |
| *p-value* |  | **0.030** | **0.015** | **0.001** | **0.000** |  |  |

Notes: Values significant at the p≤ 0.05 level are indicated in boldface. “All” indicates the combination of all datasets.

**S5 Table. Parameter estimates for the citation model of strong ties in 1993-2003.**

| Dataset | *A* | $\rho_{i,t}$ | $ln\bar{c_{i,t}}$ | $ln\bar{a_{i,t}}$ | $lnN_{i,t}$ | *N_obs._* | *Adj.R^2^* |
| --- | --- | --- | --- | --- | --- | --- | --- |
| All | 72 | 0.182 | 1.533 | 0.022 | -0.389 | 1252 | 0.20 |
| *p-value* |  | **0.014** | 0.303 | 0.980 | **0.000** |  |  |
| High-productivity | 7 | 0.632 | 3.668 | -10.855 | 0.511 | 141 | 0.10 |
| *p-value* |  | **0.034** | 0.558 | **0.000** | **0.002** |  |  |
| Low-productivity | 65 | 0.180 | 1.538 | 0.229 | -0.421 | 1111 | 0.25 |
| *p-value* |  | **0.022** | 0.330 | 0.806 | **0.000** |  |  |

Notes: Values significant at the p≤ 0.1 level are indicated in boldface. “All” indicates the combination of all datasets.

**S6 Table. Parameter estimates for the citation model of strong ties in 2004-2013.**

| Dataset | *A* | $\rho_{i,t}$ | $ln\bar{c_{i,t}}$ | $ln\bar{a_{i,t}}$ | $lnN_{i,t}$ | *N_obs._* | *Adj.R^2^* |
| --- | --- | --- | --- | --- | --- | --- | --- |
| All | 72 | 0.182 | 1.533 | 0.022 | -0.389 | 1252 | 0.20 |
| *p-value* |  | **0.014** | 0.303 | 0.980 | **0.000** |  |  |
| High-productivity | 7 | 0.632 | 3.668 | -10.855 | 0.511 | 141 | 0.10 |
| *p-value* |  | **0.034** | 0.558 | **0.000** | **0.002** |  |  |
| Low-productivity | 65 | 0.180 | 1.538 | 0.229 | -0.421 | 1111 | 0.25 |
| *p-value* |  | **0.022** | 0.330 | 0.806 | **0.000** |  |  |

Notes: Values significant at the p≤ 0.1 level are indicated in boldface. “All” indicates the combination of all datasets.


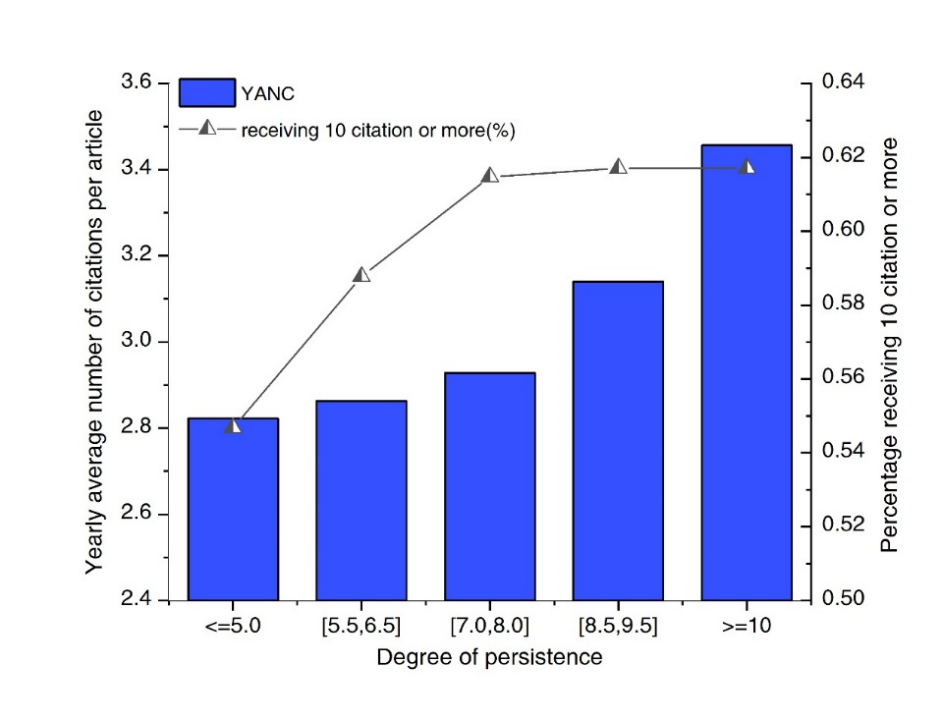


**S6 Fig.** **Yearly average number of citations (YANC) received per article and the proportion of coauthored articles that have received at least 10 citations (CAP10C) per year for different persistence groups in 1993-2003.**


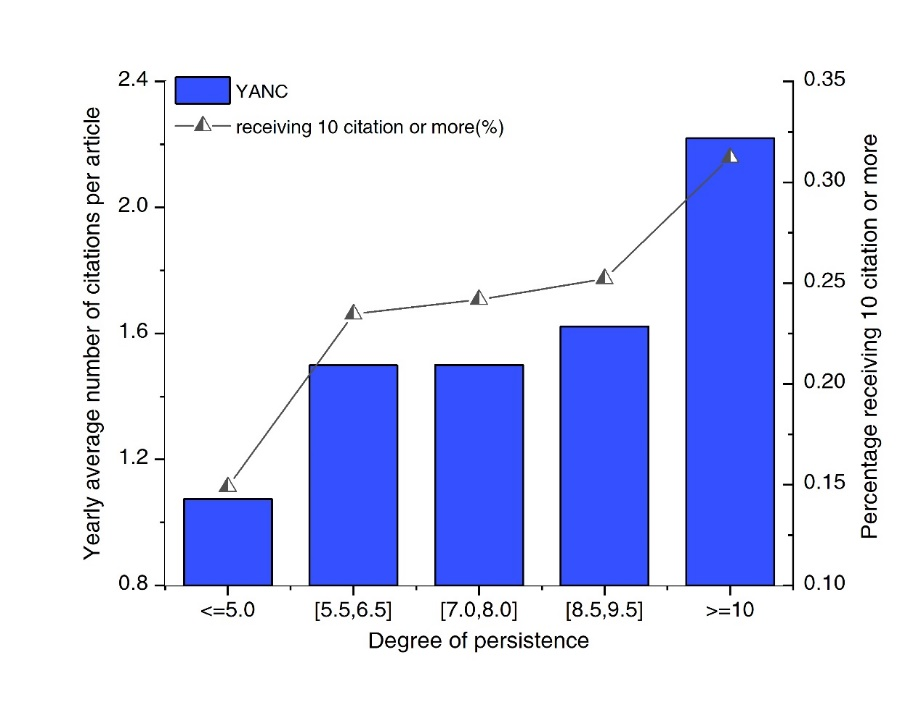


**S7 Fig.** **Yearly average number of citations (YANC) received per article and the proportion of coauthored articles that have received at least 10 citations (CAP10C) per year for different persistence groups in 2004-2013.**


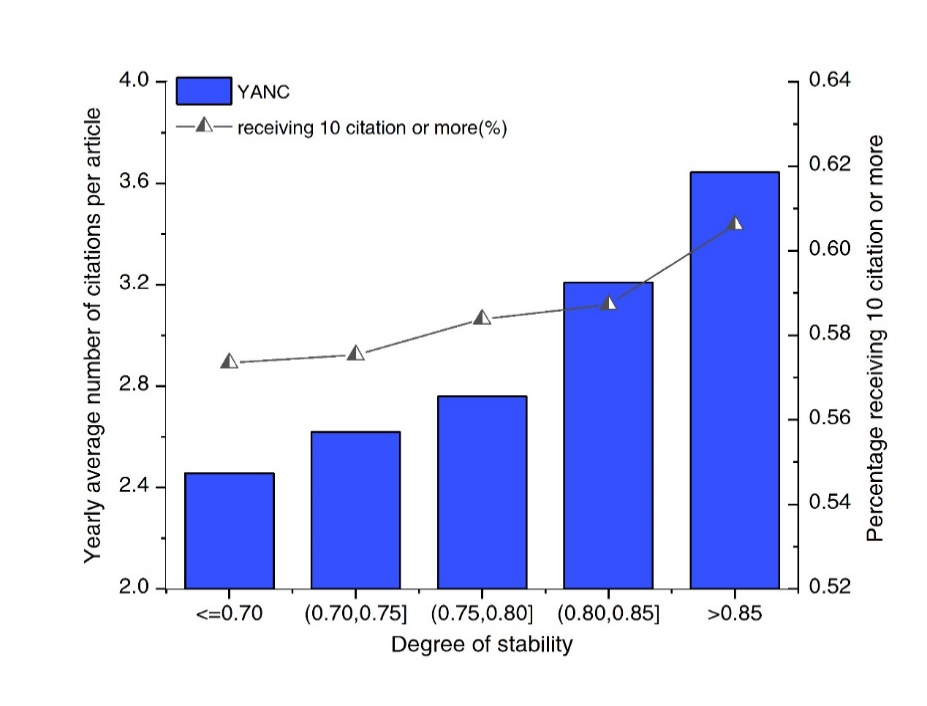


**S8 Fig.** **Yearly average number of citations (YANC) received and proportion of coauthored articles that have received at least 10 citations (CAP10C) per year for different stability groups in 1993-2003.**


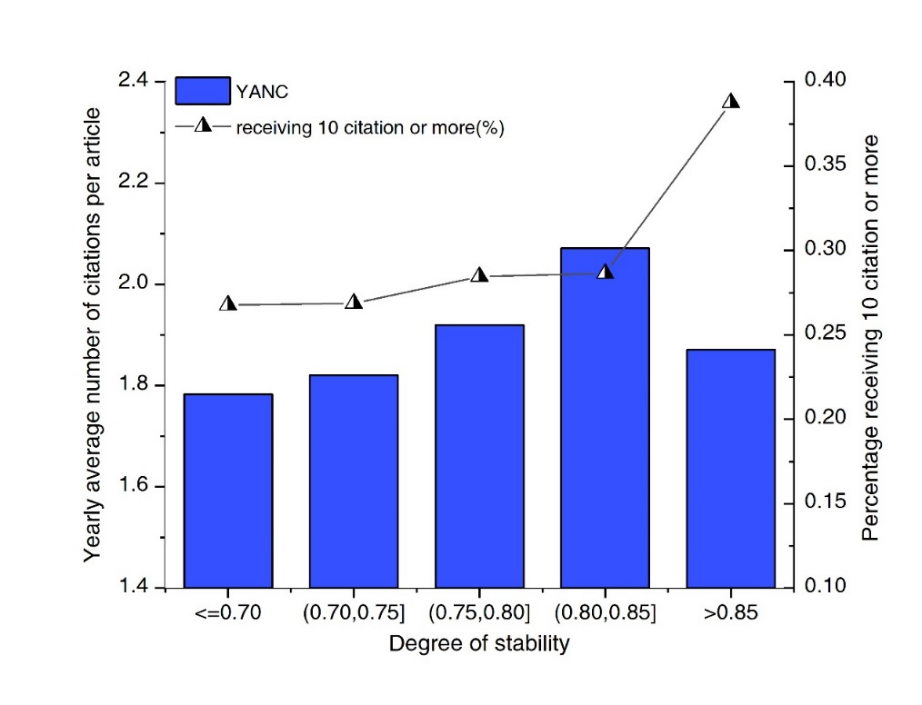


**S9 Fig.** **Yearly average number of citations (YANC) received and proportion of coauthored articles that have received at least 10 citations (CAP10C) per year for different stability groups in 2004-2013.**


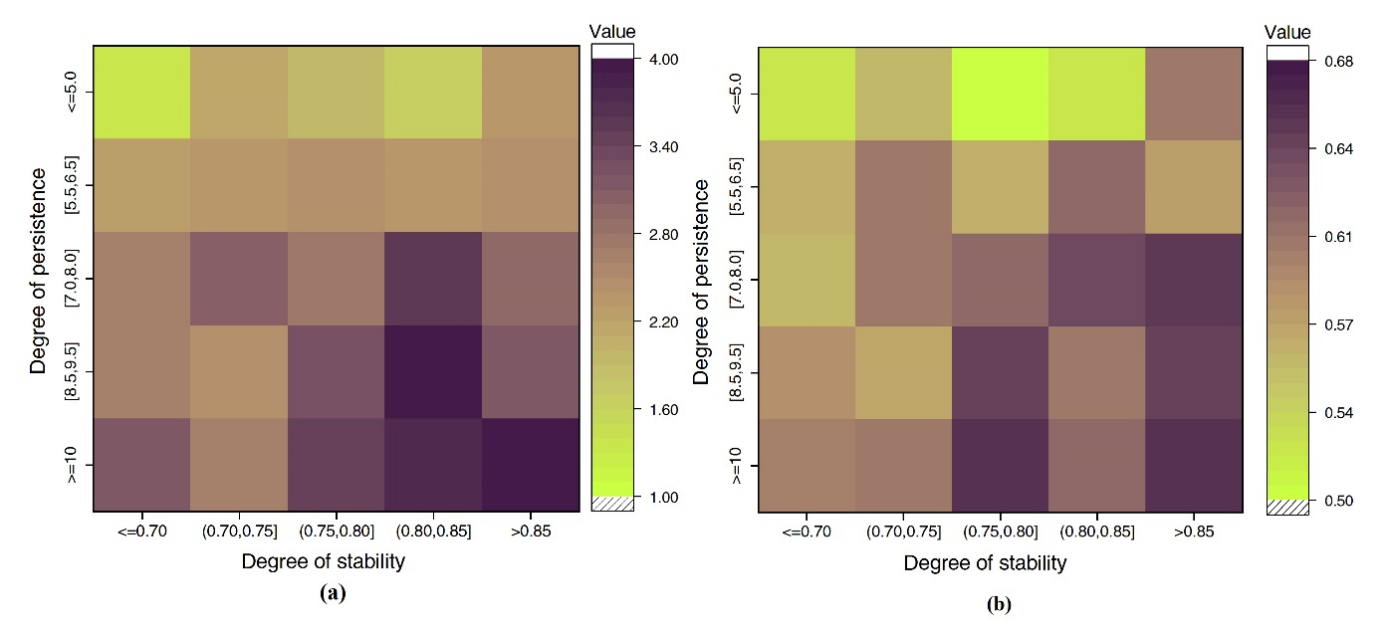


**S10 Fig. Relationships among collaboration persistence, stability and research performance from different aspects in 1993-2003.** (a) the yearly average number of citations (YANC) received and (b) the proportion of coauthored articles that have received at least 10 citations (CAP10C); shading is proportional to the value of the research performance indicators of the collaborative country pairs with the corresponding degree of stability and persistence.


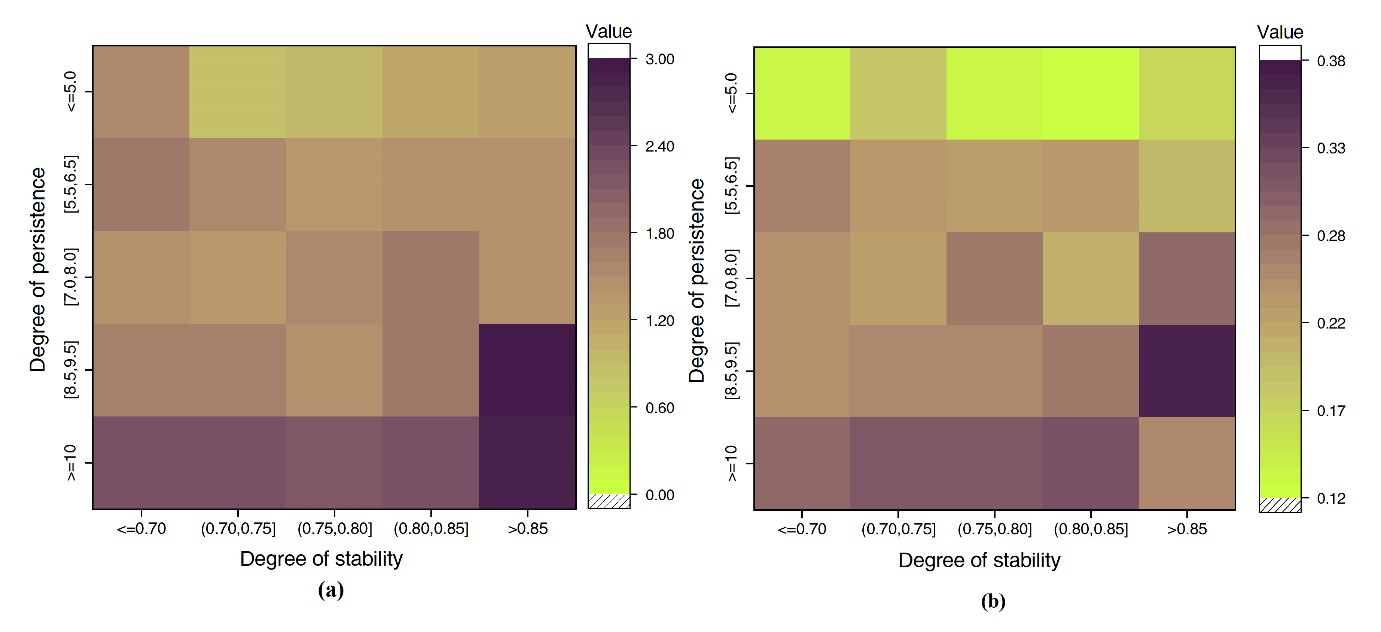


**S11 Fig. Relationships among collaboration persistence, stability and research performance from different aspects in 2004-2013.** (a) the yearly average number of citations (YANC) received and (b) the proportion of coauthored articles that have received at least 10 citations (CAP10C); shading is proportional to the value of the research performance indicators of the collaborative country pairs with the corresponding degree of stability and persistence.

**S7 Table. Parameter estimates for the productivity model of super ties in 1993-2003.**

| Dataset | *A* | $\rho_{i,t}$ | $ln\bar{c_{i,t}}$ | $ln\bar{a_{i,t}}$ | $lnN_{i,t}$ | *N_obs._* | *Adj.R^2^* |
| --- | --- | --- | --- | --- | --- | --- | --- |
| All | 72 | 0.033 | -0.781 | 0.657 | 0.528 | 1224 | 0.27 |
| *p-value* |  | **0.027** | **0.012** | **0.000** | **0.000** |  |  |
| High-productivity | 7 | 0.003 | -3.294 | 3.675 | 0.273 | 133 | 0.00 |
| *p-value* |  | 0.985 | 0.360 | **0.021** | **0.000** |  |  |
| Low-productivity | 65 | 0.033 | -0.767 | 0.603 | 0.536 | 1091 | 0.32 |
| *p-value* |  | **0.030** | **0.015** | **0.001** | **0.000** |  |  |

Notes: Values significant at the p≤ 0.05 level are indicated in boldface. “All” indicates the combination of all datasets.

**S8 Table. Parameter estimates for the productivity model of super ties in 2004-2013.**

| Dataset | *A* | *φ_t_* | $ln\bar{c_{i,t}}$ | $ln\bar{a_{i,t}}$ | $lnN_{i,t}$ | *N_obs._* | *Adj.R^2^* |
| --- | --- | --- | --- | --- | --- | --- | --- |
| All | 72 | 0.033 | -0.781 | 0.657 | 0.528 | 1224 | 0.27 |
| *p-value* |  | **0.027** | **0.012** | **0.000** | **0.000** |  |  |
| High-productivity | 7 | 0.003 | -3.294 | 3.675 | 0.273 | 133 | 0.00 |
| *p-value* |  | 0.985 | 0.360 | **0.021** | **0.000** |  |  |
| Low-productivity | 65 | 0.033 | -0.767 | 0.603 | 0.536 | 1091 | 0.32 |
| *p-value* |  | **0.030** | **0.015** | **0.001** | **0.000** |  |  |

Notes: Values significant at the p≤ 0.05 level are indicated in boldface. “All” indicates the combination of all datasets.

**S9 Table. Parameter estimates for the citation model of super ties in 1993-2003.**

| Dataset | *A* | *φ_t_* | $ln\bar{c_{i,t}}$ | $ln\bar{a_{i,t}}$ | $lnN_{i,t}$ | *N_obs._* | *Adj.R^2^* |
| --- | --- | --- | --- | --- | --- | --- | --- |
| All | 72 | 0.182 | 1.533 | 0.022 | -0.389 | 1252 | 0.20 |
| *p-value* |  | **0.014** | 0.303 | 0.980 | **0.000** |  |  |
| High-productivity | 7 | 0.632 | 3.668 | -10.855 | 0.511 | 141 | 0.10 |
| *p-value* |  | **0.034** | 0.558 | **0.000** | **0.002** |  |  |
| Low-productivity | 65 | 0.180 | 1.538 | 0.229 | -0.421 | 1111 | 0.25 |
| *p-value* |  | **0.022** | 0.330 | 0.806 | **0.000** |  |  |

Notes: Values significant at the p≤ 0.05 level are indicated in boldface. “All” indicates the combination of all datasets.

**S10 Table. Parameter estimates for the citation model of super ties in 2004-2013.**

| Dataset | *A* | *φ_t_* | $ln\bar{c_{i,t}}$ | $ln\bar{a_{i,t}}$ | $lnN_{i,t}$ | *N_obs._* | *Adj.R^2^* |
| --- | --- | --- | --- | --- | --- | --- | --- |
| All | 72 | 0.182 | 1.533 | 0.022 | -0.389 | 1252 | 0.20 |
| *p-value* |  | **0.014** | 0.303 | 0.980 | **0.000** |  |  |
| High-productivity | 7 | 0.632 | 3.668 | -10.855 | 0.511 | 141 | 0.10 |
| *p-value* |  | **0.034** | 0.558 | **0.000** | **0.002** |  |  |
| Low-productivity | 65 | 0.180 | 1.538 | 0.229 | -0.421 | 1111 | 0.25 |
| *p-value* |  | **0.022** | 0.330 | 0.806 | **0.000** |  |  |

Notes: Values significant at the p≤0.05 level are indicated in boldface. “All” indicates the combination of all datasets.

**References**

1. Petersen AM. Quantifying the impact of weak, strong, and super ties in scientific careers. Proceedings of the National Academy of Sciences. 2015; 112(34): E4671-E4680.
2. Delignette-Muller M L, Dutang C. fitdistrplus: An R package for fitting distributions[J]. Journal of statistical software. 2015; 64: 1-34.
3. Krapivsky, Pavel L., Sidney Redner, and Eli Ben-Naim. A kinetic view of statistical physics. Cambridge University Press, 2010.
